# Feasibility, acceptability, and preliminary efficacy of AdaptRehab VR, an immersive virtual reality system for upper limb stroke rehabilitation: A mixed-methods study in Ethiopia

**DOI:** 10.1371/journal.pone.0360216

**Published:** 2026-10-09

**Authors:** Chala Diriba Kenea, Teklu Gemechu Abessa, Dheeraj Lamba, Bruno Bonnechère

**Affiliations:** 1 Department of Information Science, Faculty of Computing and Informatics, Jimma Institute of Technology, Jimma University, Jimma, Oromia, Ethiopia; 2 REVAL Rehabilitation Research Center, Technology-Supported and Data-Driven Rehabilitation, Data Science Institute, Faculty of Rehabilitation Sciences, University of Hasselt, Diepenbeek, Belgium; 3 Department of Special Needs and Inclusive Education, Jimma University, Jimma, Oromia, Ethiopia; 4 Department of Physiotherapy, Faculty of Medical Sciences, Institute of Health, Jimma University, Jimma, Oromia, Ethiopia; 5 Department of PXL—Healthcare, PXL University of Applied Sciences and Arts, Hasselt, Belgium; The Hong Kong Polytechnic University, HONG KONG

## Abstract

Stroke is a leading cause of long-term disability, with a disproportionate burden in low- and middle-income countries (LMICs), where upper limb impairment emerges as one of its most prevalent and debilitating consequences. Upper limb impairment significantly limits independence and community participation, and this makes rehabilitation a critical priority. While conventional therapy is essential, its effectiveness is often limited by challenges in patient engagement and adherence. To address these barriers, immersive virtual reality (imVR) offers a promising modality for enhancing upper limb rehabilitation. This study examined the feasibility, acceptability, safety, and upper extremity outcomes of the AdaptRehab VR system in Ethiopia with the aim of informing future randomized controlled trials. A mixed-methods design was used, including quantitative assessments of upper extremity function, usability, user experience, and cybersickness, alongside qualitative interviews and focus group discussion (FGD). The study was conducted at Jimma University Teaching Hospital, Ethiopia, and enrolled 20 adults aged ≥18 years with subacute or chronic stroke. Eighteen participants completed nine AdaptRehab VR sessions over three weeks. Physiotherapists collected baseline and post-intervention data. In addition, 18 participants (interview) and 6 physiotherapists (FGD) provided qualitative feedback. The study followed CONSORT guidelines for pilot and feasibility studies. Feasibility was excellent, with 93% adherence. System Usability Scale scores indicated good usability (mean 76.2 ± 12.1). User Experience Questionnaire ratings were positive across attractiveness (1.92 ± 0.84), pragmatic quality (1.78 ± 0.79), and hedonic quality (1.84 ± 0.81). Cybersickness was minimal, with mild transient symptoms (sweating: n = 6; fatigue and headache: n = 3). Significant improvements were observed in the Action Research Arm Test (33.0 ± 13.2 to 48.6 ± 13.1, p < .001), and Box and Block Test (medians of 21.5 to 29, p < .001). Qualitative findings confirmed high acceptability, motivation, and perceived functional gains. The AdaptRehab VR system demonstrated strong feasibility, acceptability, and safety for upper limb stroke rehabilitation in Ethiopia. Positive user experiences and functional improvements support its potential in LMICs settings, warranting a fully powered randomized controlled trial.

**Trial registration:**

ClinicalTrials.gov NCT07412899

## 1. Introduction

Stroke constitutes a significant global health burden, affecting approximately fifteen million individuals annually and resulting in over 5.5 million deaths worldwide [1,2]. Notably, two-thirds of stroke-related mortality occurs in low- and middle-income countries (LMICs) [3]. This disparity is further emphasized by the concentration of over 87% of stroke-related disability-adjusted life years in LMICs, a rate approximately seven times higher than in high-income countries (HICs) [4]. This substantial burden underscores the critical importance of accessible rehabilitation services [5].

With substantial effects on physical, cognitive, and psychosocial functioning, stroke is one of the main causes of long-term disability worldwide [6]. Ethiopia had an age-standardized stroke incidence rate of 151.5 per 100,000, with ischemic stroke accounting for a large portion of incidents, according to the Global Burden of Disease study [7]. Stroke is becoming increasingly prevalent, and the related impairments and reduced quality of life constitute major public health concerns in Ethiopia [8]. Fifty to eighty percent of stroke survivors experience upper-limb impairment [9], which can significantly impact an individual’s capacity for independent living and community participation by reducing mobility, dexterity, and functional independence in everyday tasks [10]. Deficits in muscle strength, range of motion, coordination, and sensory processing characterize stroke-related upper limb impairment. These disabilities may limit an individual’s ability to perform self-care, participate fully in social and community-related work, or engage in leisure activities, leading to profound consequences [11,12]. Activities of Daily Living (ADLs) including feeding, bathing, grooming, dressing, functional mobility, toileting, and personal hygiene require effective upper limb function for maintaining independence and quality of life [13,14].

LMICs face considerable challenges in delivering equitable, high-quality rehabilitation care [15]. The most pressing concerns include a severe shortage of rehabilitation professionals, with some regions reporting as few as 0.5 therapists per 10,000 population, and inadequate infrastructure, particularly in rural areas [16]. Numerous stroke survivors in resource-constrained areas in Ethiopia, such as Jimma, Oromia, Ethiopia, fail to receive adequate interventions to regain upper limb function since comprehensive stroke rehabilitation services are scarce and healthcare professionals are insufficient in such areas [17].

In response to these challenges, various initiatives have emerged. At the global level, the World Health Organization’s 2030 Rehabilitation Initiative represents a significant effort to strengthen rehabilitation services worldwide [18]. At the continental level, evolving regional priorities are reflected in health-focused frameworks such as Africa’s Agenda 2063 [19]. At the national level, countries such as Ethiopia have integrated rehabilitation services into their healthcare frameworks in both the Health Sector Transformation Plan II and the five-year National Rehabilitation and Assistive Technology Strategic Plan [20].

Recent technological developments have catalyzed technology-supported rehabilitation innovations. Emerging technologies present potential solutions to increase rehabilitation service provision despite shortages of healthcare professionals, particularly in LMICs. Among various technologies, Virtual Reality (VR) has emerged as one of the most promising modalities for stroke rehabilitation.

Immersive VR (imVR) has demonstrated effectiveness in rehabilitation through multiple mechanisms: reducing pain, accelerating recovery times, providing realistic environments, decreasing dependence on rehabilitation personnel, enabling increased treatment intensity and frequency, facilitating creative treatment delivery, standardizing therapeutic activities, and improving overall recovery outcomes [21,22]. Its key features include real-time feedback [23], customizable and adaptive environments [24], enhanced motivation [25,26], safe and controlled environments [27], and multisensory stimulation incorporating visual, auditory, and haptic feedback [28]. Additionally, imVR facilitates essential repetitive exercises [29,30], with numerous studies demonstrating its effectiveness in stroke rehabilitation [31,32]. Furthermore, recent studies have demonstrated the feasibility of implementing advanced rehabilitation technologies in LMICs [33], highlighting their significant potential in enhancing rehabilitation services in regions facing shortages of health professionals [34].

Nevertheless, the implementation of imVR in healthcare, particularly for rehabilitation in LMICs, is constrained by several challenges, such as limited technical expertise for system development and maintenance [21], insufficient multidisciplinary collaboration [33], inadequate government funding [35], and a shortage of rehabilitation services and technologies [36–42]. Existing imVR applications primarily designed for HICs often fail to address the specific needs and technical realities of LMIC. Moreover, efforts to adapt these HICs-based applications in LMICs are frequently constrained by challenges such as cultural sensitivity issues, proprietary restrictions, and frequent technological updates. These challenges are further compounded by inadequate training and limited awareness of imVR technologies [42], limited research on effectiveness in LMIC contexts [43], and significant cultural and language barriers, as most systems are designed in English [44].

Despite the increasing stroke patient population in LMICs, there are currently no commercially available imVR solutions incorporating local cultural factors [45]. This gap is particularly critical given that 50–80% of stroke survivors experience upper limb impairment [46], which significantly limit their ADLs and contribute to perceived disability. This integrates concepts from imVR and motor learning, all of which may enhance the efficacy and delivery of rehabilitation therapies [47,48].

Therefore, the primary aim of this study was to investigate the feasibility of the AdaptRehab VR system for upper limb rehabilitation among stroke survivors in Jimma, Oromia, Ethiopia. This system, specifically co-developed for neureorehabiltiation in LMICs, includes six games (Basket Bloom, Strike Zone, TapQuest, FruitFall Frenzy, Precision Pitch, and Bean Picker Pro) each designed to target different therapeutic goals [49]. The study addresses a critical gap, as prior research on this specific intervention technique remains limited, particularly in relation to the sociocultural health beliefs of the target population. The absence of data on this intervention in the resources-limited context renders this study a significant contribution to the field. By evaluating the feasibility, acceptability, enjoyment, and safety of this intervention, the study will provide essential insights to guide the design and execution of a larger-scale randomized controlled trial [50]. The primary objectives were to investigate the feasibility and acceptability of the proposed AdaptRehab VR system for upper limb stroke rehabilitation in resource limited-settings.

## 2. Materials and methods

### 2.1 Study design

A mixed-methods design incorporating both quantitative and qualitative data was used. Quantitative data were collected from participants and included measures of upper extremity outcomes, usability, user experience, and cybersickness. Complementing these assessments, qualitative insights were obtained through participant interviews and focus group discussion (FGD) with physiotherapists. The methodology and reporting of this non-randomized pilot feasibility study adhered to the CONSORT extension for pilot and feasibility studies together with the TREND Statement reporting guidelines [51–53]. The study was conducted at the physiotherapy center of Jimma University Teaching Hospital (JUTH) in Jimma, Oromia, Ethiopia.

Study participants included stroke survivors with upper limb motor impairments and physiotherapists. Stroke survivors undergoing rehabilitation at JUTH were considered for recruitment. Following medical record review and eligibility assessment, adults aged > 18 years presenting with subacute or chronic stroke [54], who provided consent for research contact were recruited. Physiotherapists rated their satisfaction with intervention sessions and documented any adverse events experienced during participation. All physiotherapists teaching at department of Physiotherapy at JUTH were invited to provide feedback on the AdaptRehab VR system, evaluating its suitability and acceptability for upper limb rehabilitation in stroke survivors. The sample comprised 18 stroke survivors. Additionally, based on their consent, all six physiotherapists teaching at department of Physiotherapy at JUTH were included [55].

### 2.2 Protocol adherence and deviations

The study was conducted in accordance with the approved study protocol reviewed and approved by the Institutional Review Board (IRB) of Jimma University (JUIH/IRB/0479/25; approval date: 12 April 2025) and registered as a clinical trial (NCT07412899). The study procedures, including the study design, participant eligibility criteria, AdaptRehab VR intervention, and outcome assessments, were implemented as described in the predefined protocol.

### 2.3 Eligibility criteria

The following inclusion criteria were applied: adults aged 18 years or older with a confirmed diagnosis of ischemic or hemorrhagic stroke, a stroke onset of three months or more prior to enrollments, who were in the subacute or chronic stage of recovery, and who presented with upper limb impairment [56–58]. Participants were required to be medically stable, capable of understanding and following instructions, and to possess adequate cognitive and visual abilities to safely interact with the AdaptRehab VR system. Exclusion criteria comprised a diagnosis of severe cognitive impairment, significant visual impairment interfering with AdaptRehab VR interaction, the presence of severe psychological or psychiatric disorders, and any medical condition that could compromise safe participation in AdaptRehab VR based rehabilitation.

### 2.4 Intervention

The AdaptRehab VR system was used and tested for upper limb rehabilitation. This system was previously developed by the authors with specific LMICs rehabilitation’s needs and requirements; complete descriptions and the co-creation process are presented elsewhere [31]. AdaptRehab VR activities were customized to each participant’s individual needs and treatment plan. Throughout the intervention, participants received guidance and assistance from qualified physiotherapists, with the AdaptRehab VR system installed in a designated area within the JUTH Physiotherapy clinic. Participants were encouraged, as appropriate, to practice with the AdaptRehab VR system independently between supervised sessions. Its intervention intensity was standardized across participants (9 sessions over 3 weeks, approximately 30 minutes per session). But variations in access to concurrent rehabilitation services may have influenced overall rehabilitation exposure and outcomes.

At baseline and post-intervention, upper limb motor function was assessed using the Action Research Arm Test (ARAT) and the Box and Block Test (BBT). In addition, face-to-face interviews and standardized questionnaires were used to collect both quantitative and qualitative data from stroke survivors. Furthermore, FGD were conducted with physiotherapists to explore their experiences and perceptions of the intervention.

As this was a feasibility study, participants were not restricted from receiving concurrent therapies during the study period, including conventional rehabilitation and routine stroke-related medical care. However, the specific types, frequencies, and durations of these additional interventions were not prospectively recorded.

Feasibility progression criteria were defined a priori based on recommendations for pilot and feasibility studies [48,59]. Criteria included recruitment, retention, adherence, usability, and safety outcomes. Progression to a larger trial was considered feasible if the intervention demonstrated acceptable retention (≥80%), adherence (≥80% of sessions), usability (SUS ≥ 70), and no serious intervention-related adverse events [60].

### 2.5 Outcome measures

#### 2.5.1 Primary outcomes.

Primary outcomes were usability (assessed using the System Usability Scale [SUS]), user experience (assessed using User Experience Questionnaire [UEQ]), cybersickness (assessed using Simulator Sickness Questionnaire [SSQ]) and acceptability and satisfaction (assessed using Customer Satisfaction Score [CSS] and Net Promoter Score [NPS]), FGD and in-depth interviews.

**Recruitment, attrition, and adherence:** Recruitment rates were calculated as the number of participants approached, excluded, or agreeing to participate. Attrition rates reflected participants who withdrew or were lost to follow-up, as opposed to those who completed the study. Adherence rates were determined as the proportion of participants completing the intervention as guided, calculated from the ratio of sessions performed to prescribed sessions (9 sessions over 3 weeks).

**System Usability Scale (SUS):** The SUS was administered post-intervention by physiotherapists. The SUS comprises 10 items with 5 Likert-scale response options (1 = Strongly Disagree to 5 = Strongly Agree), providing a measure of AdaptRehab VR system usability. SUS total scores range from 0–100, with scores >68 considered above average [61].

**User Experience Questionnaire (UEQ):** The UEQ was also administered post-intervention by physiotherapists. The UEQ employs a 7-point semantic differential scale ranging from 1 (extremely negative) to 7 (extremely positive) across 26 items. Items are organized into six scales: Attractiveness, perspicuity, efficiency, dependability, stimulation, and novelty, which aggregate into three broader dimensions: Attractiveness, Pragmatic Quality (Perspicuity, Efficiency, Dependability), and Hedonic Quality (Stimulation, Novelty) [62,63]. The values mean > 0.8 represent a positive evaluation and values mean < −0.8 represent a negative evaluation [64].

**Simulator Sickness Questionnaire (SSQ):** The SSQ was administered post-intervention, with physiotherapists rating symptom severity (0 = not at all, 1 = mild, 2 = moderate, 3 = severe) across 16 items. Symptoms include general discomfort, fatigue, headache, eyestrain, increased salivation, sweating, nausea, fullness of head, blurred vision, dizziness (eyes open and closed), vertigo, stomach awareness, and burping. Total severity scores and subscale scores (Nausea, Oculomotor, Disorientation) were calculated following standard procedures [65].

**Acceptability and satisfaction:** Participant satisfaction and willingness to use the intervention in the future were assessed using Customer Satisfaction Score (CSS) and Net Promoter Score (NPS), supplemented by FGD with physiotherapists and in-depth interviews with participants. Participants scoring 9–10 were considered promoters (participants who would recommend the system), those scoring 7–8 were classified as passives (participants who were satisfied but unlikely to actively recommend the system), and those scoring 0–6 were classified as detractors (participants who were unlikely to recommend the system).

#### 2.5.2 Secondary outcomes.

For preliminary evaluation of the clinical efficacy two clinical tests were used: the Action Research Arm Test (ARAT) and the Box and Block Test (BBT). The A RAT assesses upper extremity function through four subtests: Grasp (0–18), Grip (0–12), Pinch (0–18), and Gross Movement (0–9), with a total score ranging from 0–57, where higher scores indicate better hand dexterity. The BBT measures manual dexterity by counting the number of blocks transferred from one compartment to another within 60 seconds. Higher counts indicate better hand and arm dexterity. The ARAT and BBT assessments were conducted by assessors who were independent of the intervention delivery. However, due to staffing limitations, the assessors were not blinded to participants’ study participation.

### 2.6 Data collection procedures

Prior to data collection, training was provided to the three physiotherapists involved in data collection. All quantitative data were collected from stroke survivors, while qualitative data were gathered from both stroke survivors who completed the sessions and six physiotherapists after they had been exposed to the AdaptRehab VR system.

### 2.7 Data analysis

#### 2.7.1 Quantitative analysis.

Descriptive statistics (frequencies, percentages, means, standard deviations) were used to present sociodemographic and clinical characteristics as well as primary outcomes. Secondary outcomes were analyzed using paired sample t-tests or Wilcoxon test depending on the distribution of the data. Data were entered into EpiData version 3.1 and analyzed using R Studio (version 4.2.1).

Missing data were manually reviewed prior to analysis, and only participants with available baseline and post-intervention assessments were included in the outcome analyses. Therefore, the outcome analyses were conducted using complete-case data. No post-intervention outcome data were missing among participants who completed the study; thus, multiple imputation was not required.

In addition, in light of the feasibility design and small sample size, all analyses were considered exploratory. Effect sizes and 95% confidence intervals were reported alongside *p*-values to facilitate the interpretation of preliminary intervention effects.

Bootstrap resampling procedures were applied to key feasibility outcomes to quantify uncertainty around feasibility estimates. Bootstrap confidence intervals (95%) were generated by repeatedly resampling participant-level data with replacement and calculating the distribution of the parameter estimates. This approach was selected because of the small sample size and its ability to provide robust estimates of uncertainty without relying on strict distributional assumptions.

For the SUS, UEQ and SSQ analysis the following methodology was used. SUS scores were calculated using standard procedures: for odd-numbered items (1,3,5,7,9), subtract 1 from the response; for even-numbered items (2,4,6,8,10), subtract the response from 5. Sum these values and multiply by 2.5 to obtain the total SUS score (range 0–100) [61].

UEQ data were analyzed using the UEQ Data Analysis Tool (version 12) and custom R scripts. For each item, responses were converted to values ranging from −3 to +3. Scale means were calculated by averaging relevant items, with values between −0.8 and 0.8 representing neutral evaluation, > 0.8 representing positive evaluation, and <−0.8 representing negative evaluation [62].

SSQ total severity scores and subscale scores (Nausea, Oculomotor, Disorientation) were calculated following Kennedy et al.‘s [65] weighting procedure. Subscale scores were computed by summing symptom scores within each category and multiplying by respective weighting factors.

#### 2.7.2 Qualitative analysis.

Thematic analysis was employed for in-depth interview and FGD data. Transcripts were systematically reviewed to identify recurring themes and patterns, involving systematic coding of textual information to categorize and organize key ideas and perspectives. Coded data were further explored to identify key themes capturing core participant experiences. Divergent or contradictory themes were examined to gain refined understanding. MAXQDA software facilitated qualitative data analysis.

### 2.8 Ethical considerations

Participants were treated according to the principles of justice, beneficence, and respect for humans as stated in the Declaration of Helsinki, following Good Clinical Practice and institutional and international guidelines [23]. All stroke survivors meeting inclusion criteria were fully eligible regardless of gender, ethnic background, or minority status. Following consent, participants were informed verbally and in writing that they could decline participation or withdraw at any time without negative consequences. The Institutional Review Board at Jimma University examined and approved the study protocol (JUIH/IRB/0479/25) and date of Approval: 12/04/2025, with clinical trial registration (NCT07412899). The recruitment period for this study started on 1 September 2025 and ended on 10 October 2025. Written informed consent was obtained from all participants before participation in the study. Participants were informed about the purpose of the study, study procedures, potential risks and benefits, confidentiality, and their right to withdraw from the study at any time without penalty. Participants were informed of their right to withdraw at any time. All participant data remained confidential, and adverse events were promptly reported and addressed. The data were accessed for research purposes from 1 September 2025 to 31 October 2025. The authors did not have access to information that could identify individual participants during or after data collection.

## 3. Results

### 3.1 Participant and clinical characteristics

Thirty-one stroke survivors were screened, 9 were excluded due to transportation challenges. These 9 stroke survivors resided in rural areas and lacked both local accommodation and social support necessary to remain near the hospital throughout the rehabilitation program. Transportation and accessibility barriers are well documented determinants of poor participation in outpatient rehabilitation programs. Two patients were medically unstable and therefore did not meet the eligibility criteria for participation, resulting in an overall recruitment rate of 65%. Fig 1 presents the CONSORT flow diagram summarizing participant recruitment, allocation, follow-up, and analysis.

**Fig 1:**
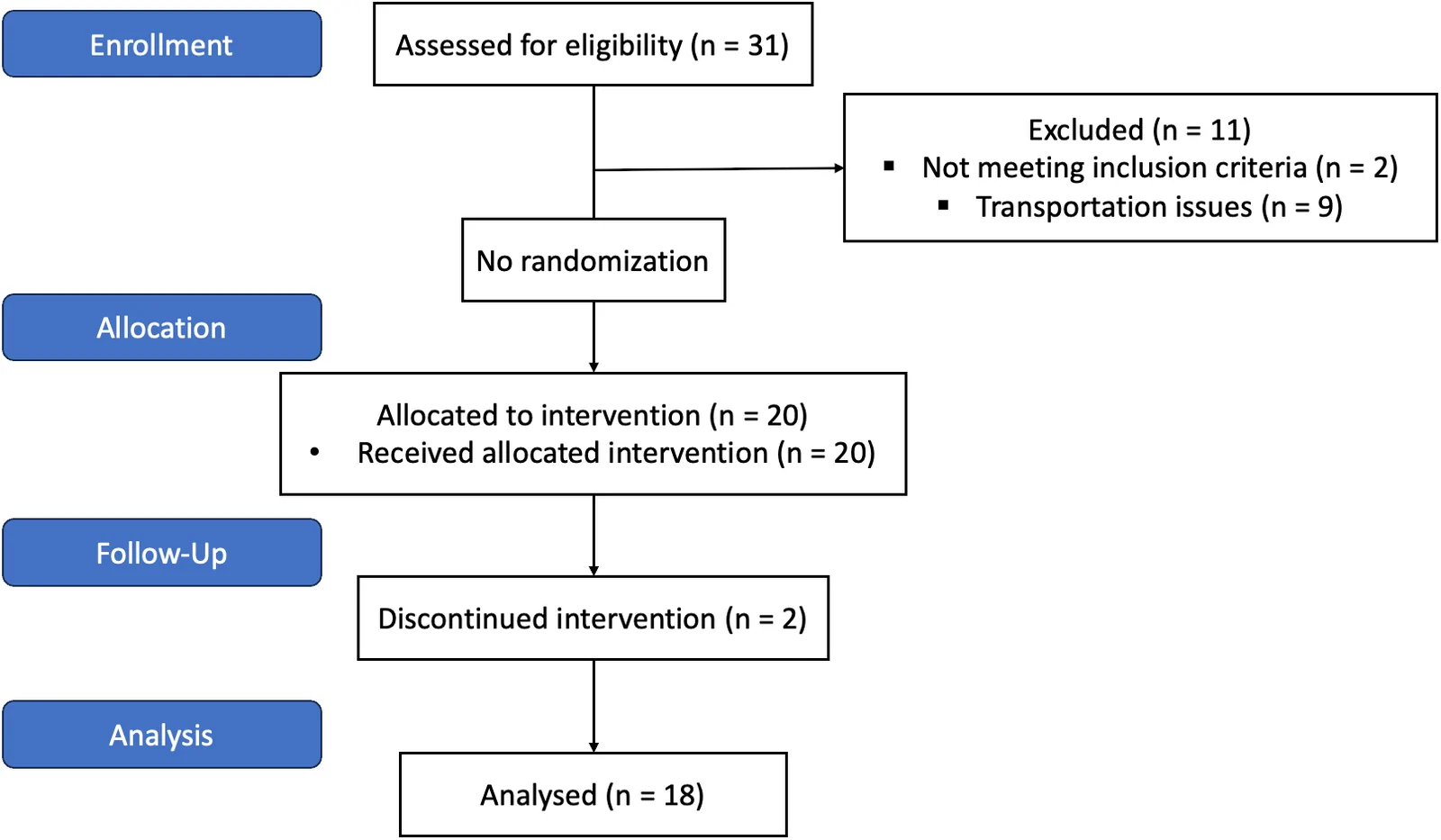
CONSORT flowchart.

The study included 20 stroke survivors with a mean age of 50.85 years (SD = 11.94). The majority were male (85%, n = 17), married (75%, n = 15), and resided in urban areas (80%, n = 16). Regarding educational status, 40% (n = 7) had completed secondary school, while 27.8% (n = 5) had completed college or above, and a further 30% (n = 6) had only primary education. Most participants were unemployed (75%, n = 13). The religious affiliation of the majority was Orthodox Christian (55%, n = 11), followed by Muslim (35%, n = 7). Regarding clinical characteristics, the mean time since stroke was 14.85 months (SD = 18.16). The most common type was ischemic stroke (75%, n = 15), and the left hemisphere was the most frequently affected brain site (65%, n = 13). The predominant physical impairment involved both the upper and lower limbs (85%, n = 17). Hypertension was the most prevalent comorbidity, present in 85% (n = 17) of participants. The majority of participants were in the chronic stage of stroke recovery (65%, n = 13). The median score for ARAT was 33 [p25 = 25, p75 = 43.75], and for BBT was 21.5 [p25 = 9.75, p75 = 28.25]. The individual characteristics are presented in Table 1.

**Table 1:** Clinical characteristics of stroke participants.

Patient ID	Type of stroke	Affected site	Other comorbidities	Stage of stroke	Since the stroke (months)	ARAT	BBT
P01	Ischemic	Left	Hypertension	Subacute	3	33	29
P02	Ischemic	Left	Hypertension	Subacute	4	15	0
P03	Ischemic	Left	Hypertension	Chronic	13	35	23
P04	Ischemic	Left	Hypertension	Chronic	20	3	0
P05	Hemorrhagic	Left	Hypertension	Subacute	5	33	12
P06	Ischemic	Both	Hypertension	Chronic	8	43	72
P07	Ischemic	Right	Hypertension	Subacute	4	39	22
P08	Ischemic	Left	Hypertension	Chronic	28	34	20
P09	Hemorrhagic	Both	Diabetes mellitus	Chronic	8	39	41
P10	Ischemic	Right	Diabetes mellitus	Chronic	12	24	23
P11	Hemorrhagic	Right	Hypertension	Subacute	3	28	21
P12	Hemorrhagic	Right	Hypertension	Subacute	5	44	19
P13	Ischemic	Left	Hypertension, Diabetes	Chronic	18	50	36
P14	Hemorrhagic	Left	Hypertension	Subacute	3	29	9
P15	Ischemic	Left	Hypertension	Chronic	20	54	34
P16	Ischemic	Left	Hypertension	Chronic	9	45	25
P17	Ischemic	Left	Hypertension	Chronic	34	14	9
P18	Ischemic	Left	Hypertension	Chronic	84	32	19
P19	Ischemic	Right	Hypertension	Chronic	7	31	26
P20	Ischemic	Right	Hypertension, Diabetes	Chronic	9	11	2

### 3.2 Intervention adherence and safety

Out of the 20 patients, 2 stopped before completing the 9 sessions, representing an attrition rate of 10%, resulting in 18 participants completing the post-intervention assessment. Outcome analyses were therefore conducted using data from the 18 participants with complete baseline and post-intervention measurements. P19 withdrew due to a lack of support to go to the clinics, including financial, family, and material support after one week (3 sessions completed). P20 discontinued due to cognitive impairments that substantially limited the capacity to understand and engage with the intervention. Specifically, he was unable to follow task instructions and consistently demonstrated non-engagement, characterized by sitting and laughing after donning the headset without participating in the exercises. Although initial exposure to the Adapt VR system was attempted during the first three sessions, meaningful interaction was not achieved, as task comprehension remained inadequate despite repeated guidance from both the physiotherapists and the system. Notably, pre-intervention cognitive screening was not conducted, which may have limited the ability to identify individuals at risk of poor engagement due to cognitive difficulties.

All the 18 participants fully completed the intervention, attending nine sessions over three weeks with each session lasting approximately 30 minutes. The overall adherence to scheduled sessions is 93%, indicating high feasibility and acceptability. Minor adverse events were reported in a few participants, these events were temporary, self-limiting, and did not interrupt or prevent the completion of the sessions (see here below).

### 3.3 Primary outcomes: Usability and adverse events

#### 3.3.1 System Usability Scale.

The mean SUS total score was 76.2 ± 12.1 (range: 52.5–92.5), indicating good usability above the commonly accepted threshold of 68. The 95% confidence interval (CI) for the mean SUS score was 69.8–83.0, while the 95% bootstrap CI was 69.8–81.7, demonstrating the precision and robustness of the usability estimate [61], Fig 2A. Individual item analysis revealed particularly high ratings for ease of use (Q3: 4.61 ± 0.61) and confidence (Q9: 4.50 ± 0.71), while perceptions of complexity (Q2: 1.78 ± 1.26) and cumbersomeness (Q8: 1.67 ± 1.03) were low. Individual results are presented Fig 2B.

**Fig 2:**
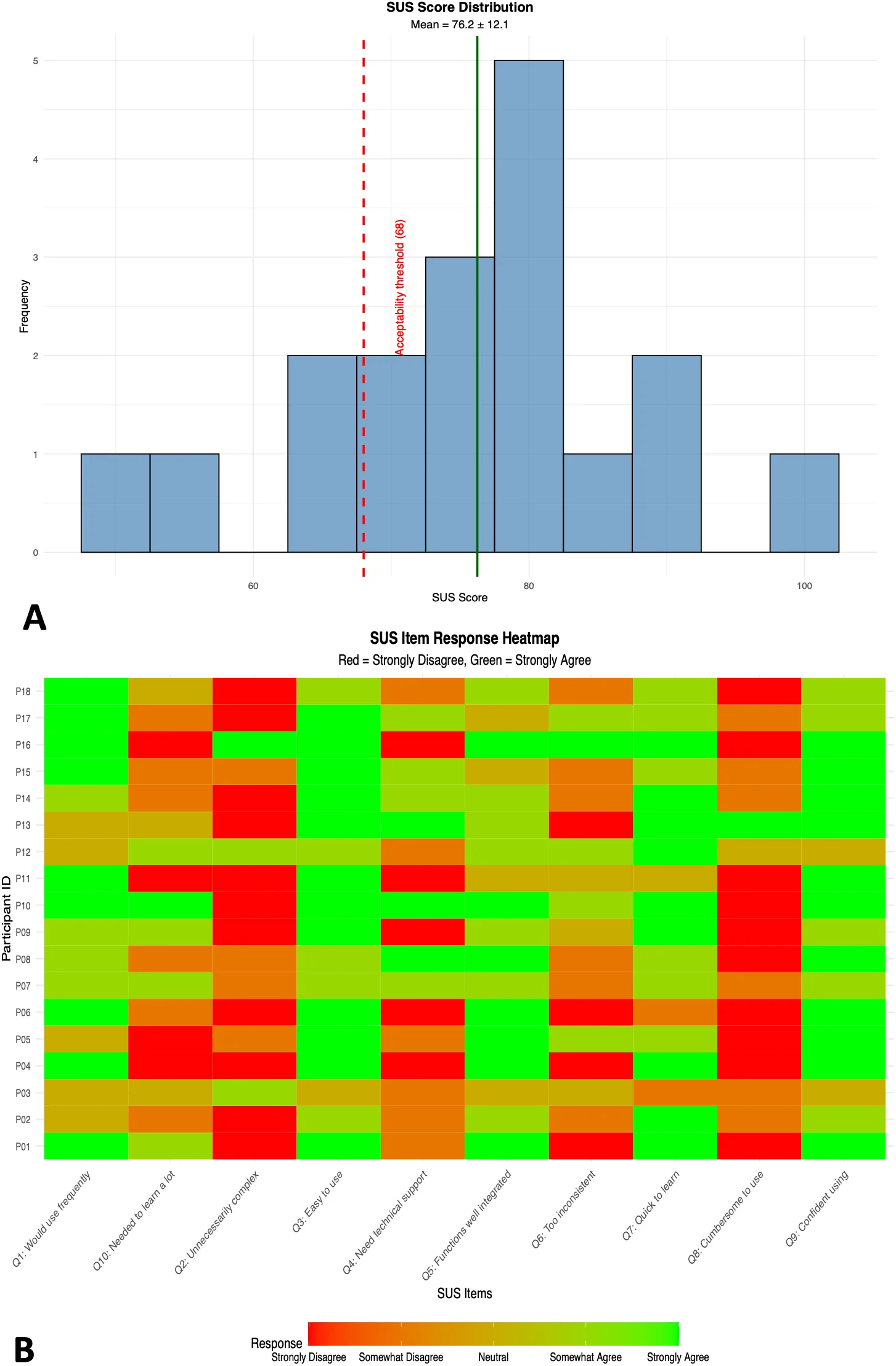
System Usability Scale (SUS) score distribution and acceptability threshold (A) and item response heatmap (B).

#### 3.3.2 User Experience Questionnaire.

All UEQ scales demonstrated positive evaluations (means >0.8). The attractiveness score was 1.92 ± 0.84, indicating a positive user experience. The 95% CI for the mean score was 1.56–2.22, while the 95% bootstrap CI was 1.59–2.18. The Pragmatic Quality score (Perspicuity, Efficiency, and Dependability) was 1.78 ± 0.79, with a 95% CI of 1.34–2.04 and a 95% bootstrap CI of 1.43–1.96. The Hedonic Quality score (Stimulation and Novelty) was 1.84 ± 0.82, with a 95% CI of 1.33–2.15 and a 95% bootstrap CI of 1.39–2.09 were both rated positively, indicating that participants found the system both usable and engaging. Complete results are presented in Fig 3.

**Fig 3:**
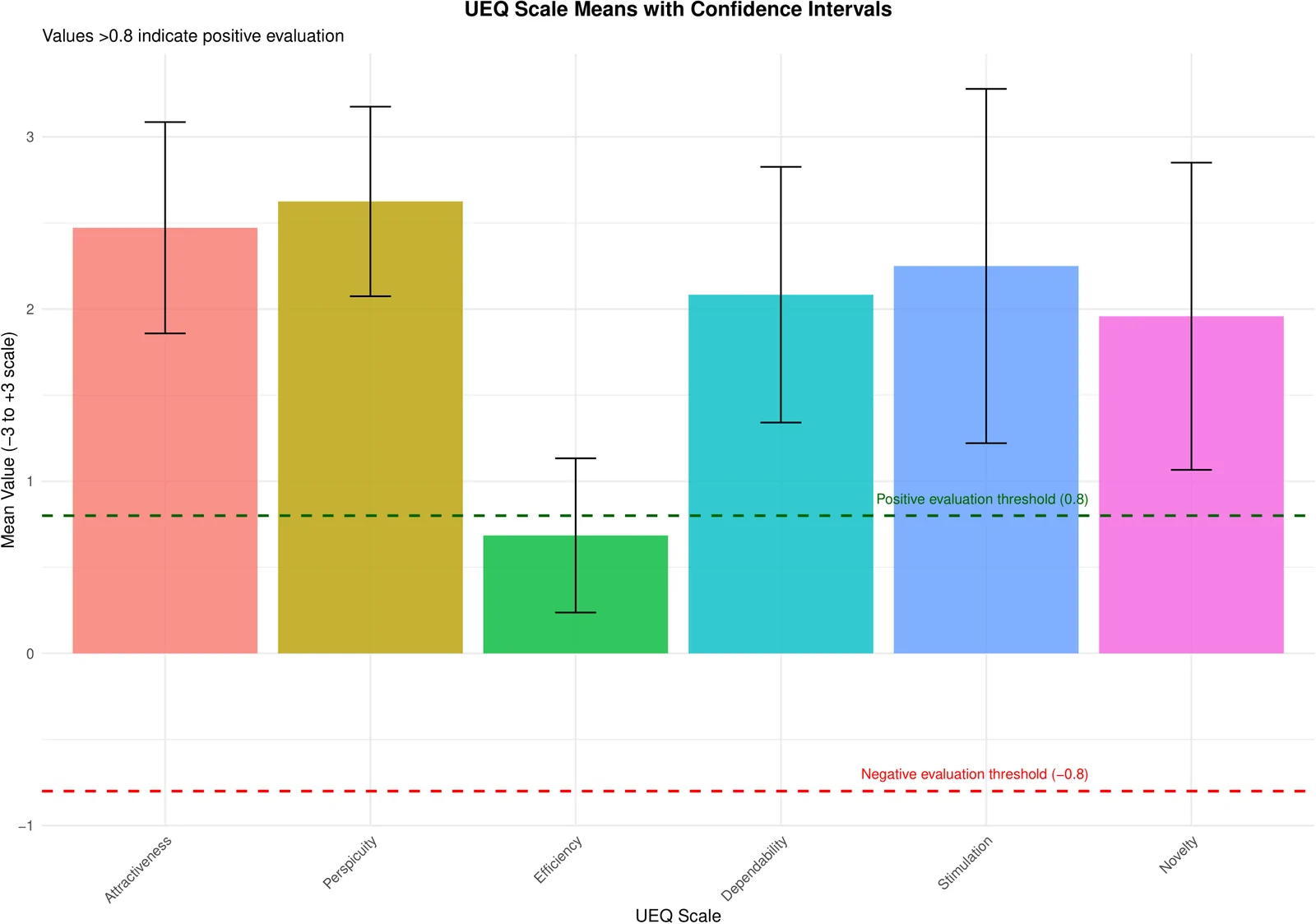
User Experience Questionnaire per categories.

#### 3.3.3 Simulator Sickness Questionnaire.

Overall, participants reported minimal simulator-related symptoms, indicating excellent system tolerability. The most frequently reported symptom was sweating (33.3%, all mild), followed by fatigue and headache (16.7% each). No moderate or severe symptoms were reported. Total severity score: 6.8 ± 4.2; Nausea subscale: 4.2 ± 3.1; Oculomotor subscale: 5.1 ± 3.8; Disorientation subscale: 3.9 ± 2.9. All scores were well below clinically significant thresholds, indicating excellent tolerability [65]. Heatmap presenting the different reported adverse events is presented in Fig 4.

**Fig 4:**
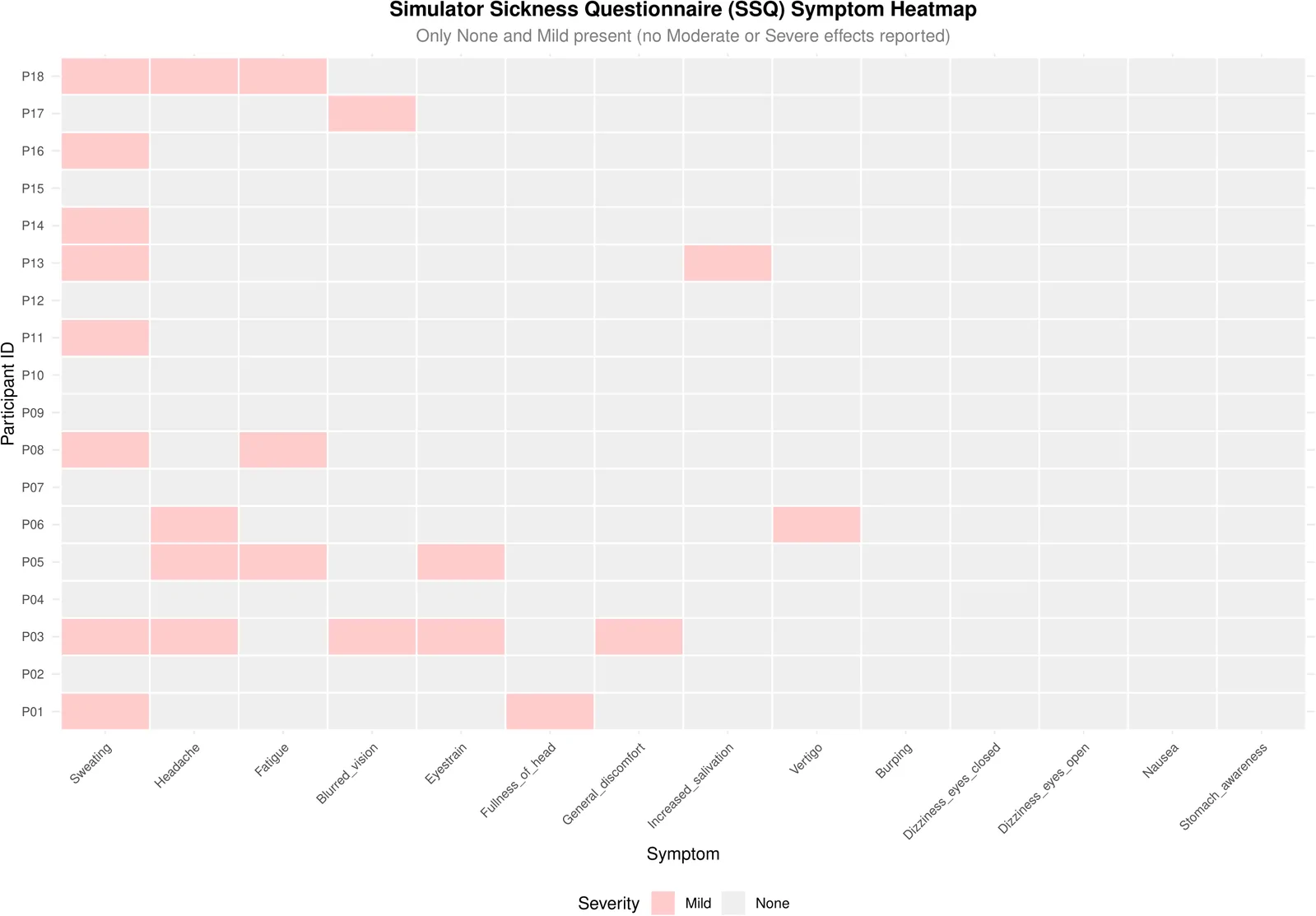
Simulator Sickness Questionnaire symptom heatmap.

#### 3.3.4 Acceptability and satisfaction.

The CSS was 89%, indicating that the vast majority of participants were satisfied with the system. The NPS was calculated as 50, derived from 9 promoters, 9 passives, and 0 detractors (Fig 5). This positive NPS reflects a strong willingness among participants to recommend the system to others, further supporting the high acceptability of the intervention.

**Fig 5:**
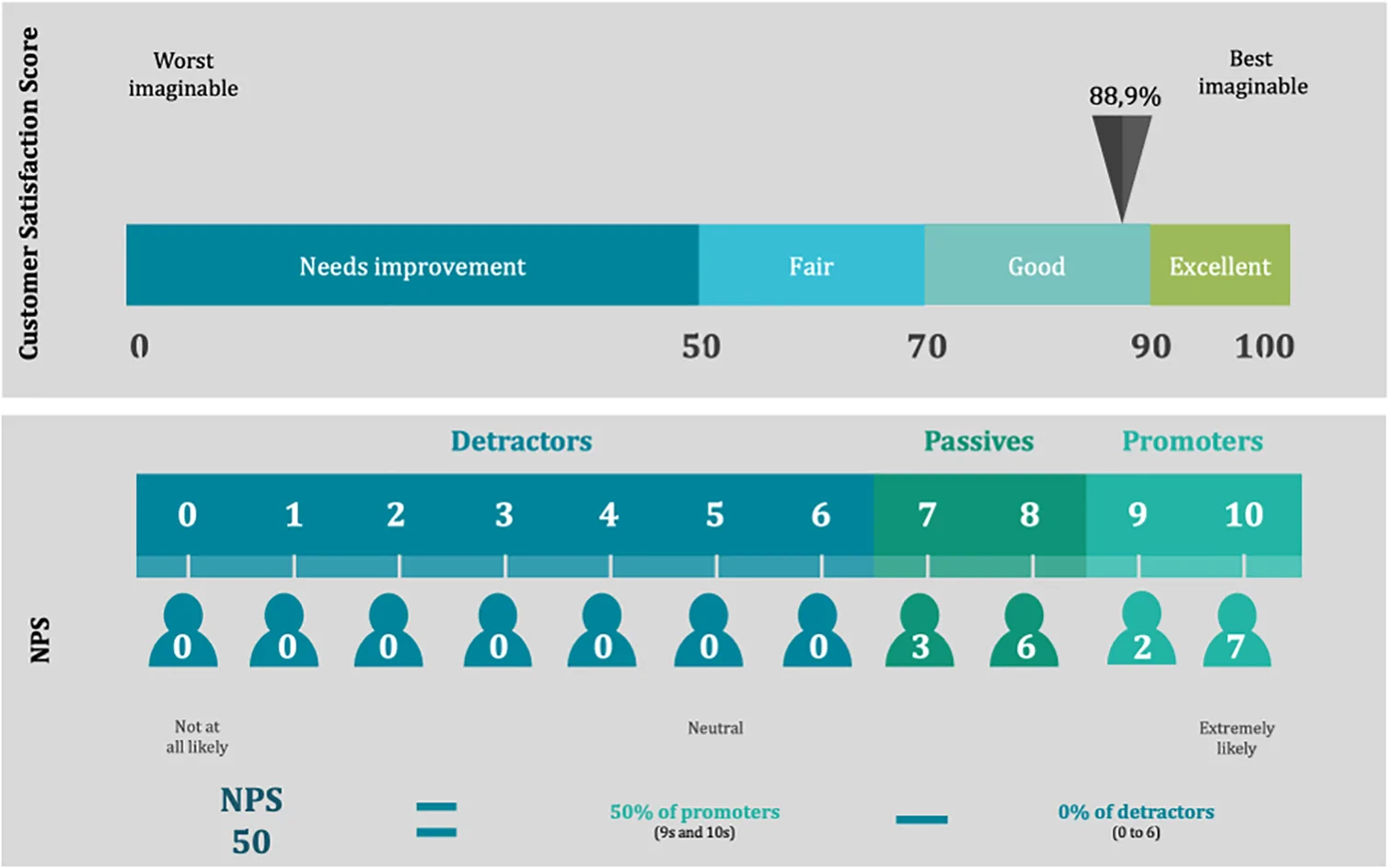
CSS and NPS scores.

### 3.4 Secondary outcomes: Upper limb functional improvements

All outcome measures demonstrated statistically significant improvements from pre- to post-intervention, with large effect sizes (Cohen’s d ranging from 0.66 to 2.90). The complete results are presented in Table 2. The ARAT total score increased from 33.0 ± 13.2 to 48.6 ± 13.1 (p < .001), and BBT scores improved by a median of 7 blocks.

**Table 2:** Pre‑ and post‑intervention upper limb functional outcomes.

Outcome Measure	Post-test Mean ± SD	Pre-test Mean ± SD	Mean Difference [95% CI]	p-value	Cohen’s d
**ARAT**					
Grasp	16.33 ± 4.52	11.61 ± 4.66	4.72 [3.13-6.33]	<.001	1.48
Grip	9.94 ± 3.37	6.89 ± 2.97	3.05 [2.21-3.90]	<.001	1.80
Pinch	14.28 ± 6.15	8.78 ± 5.16	5.50 [3.35-7.65]	<.001	1.27
Gross Movement	8.06 ± 1.66	5.72 ± 1.93	2.34 [1.77-2.90]	<.001	2.05
**BBT**					
Time	21.5 (p25 = 11.25; p75 = 30.25)	29 (p25 = 20; p75 = 45)	7 (p25 = 3.75; p75 = 10.25)	<.001	NA

Values are mean ± standard deviation. Paired sample t‑tests revealed significant improvements across all ARAT subscales and BBT scores (all p < 0.001). Effect sizes (Cohen’s d) indicate large to very large clinical effects.

### 3.5 Qualitative findings

#### 3.5.1 Participant interviews.

Six key themes have been identified through the different interviews. The key (sub)concepts are presented in Fig 6.

**Fig 6:**
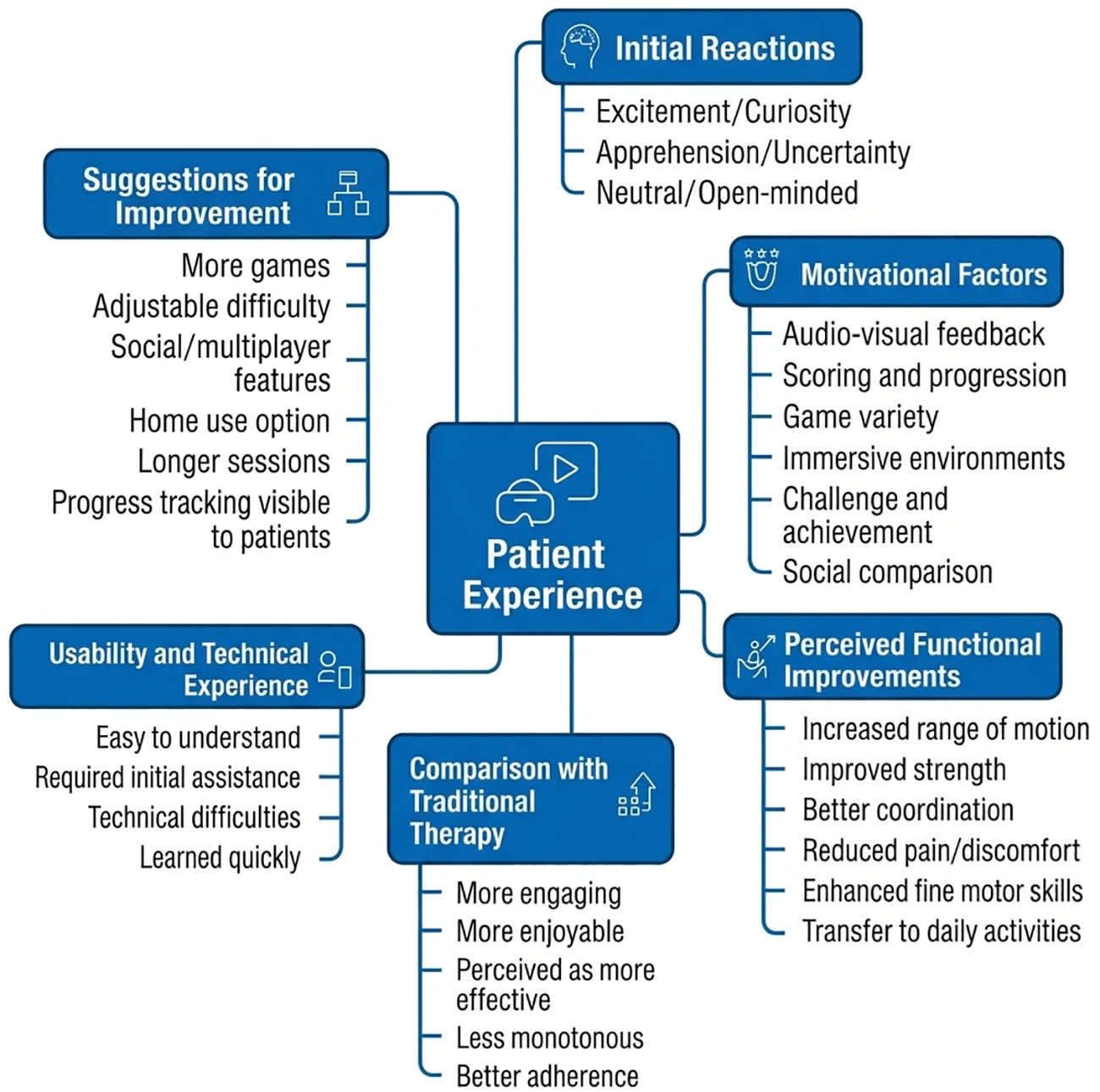
Key concepts of the thematic analysis for patients.

**Theme 1: Initial feelings and expectations:** Analysis of participants’ initial responses indicated a predominantly positive reception to the AdaptRehab VR system. Most participants (15/18, 83.3%) reported feeling excited or curious upon first being introduced to the technology. Two participants (11.1%) initially experienced apprehension; however, these concerns resolved after the first intervention session. One participant (5.6%) reported a neutral initial response.

**Theme 2: Enhanced motivation and engagement:** Participants consistently reported that the gamified nature of the intervention was a powerful driver of their motivation to engage in rehabilitation. The immersive and interactive features of the AdaptRehab VR system were consistently identified as key elements that enhanced participants’ rehabilitation experience. In particular, participants highlighted the motivating effect of the real-time multimodal feedback, including audio-visual cues, performance scoring, and progression through the games. As one participant explained, *“The sounds and colors when I caught the fruit made me want to keep playing.”* This was reinforced by a sense of achievement and progression, as another noted, *“Seeing my score increase each time motivated me to try harder.”* The game variety of activities was also crucial in maintaining interest over time; participants appreciated that the *“different games kept it interesting; I never got bored.”* Furthermore, the quality of the immersive environments was so compelling that one individual remarked, *“I forgot I was in the hospital - I felt like I was really in the AdaptRehab VR system.”* This powerful sense of presence and enjoyment directly contributed to a higher willingness to participate in therapy sessions. Participants also reported that progressively challenging exercise levels, achievement-based rewards, and opportunities for social comparison enhanced motivation and encouraged sustained engagement with the AdaptRehab VR intervention. Comparing their scores and progress with those of other participants fostered a sense of healthy competition and encouraged them to improve their performance across sessions.

**Theme 3: Perceived functional improvements:** The majority of participants (80%) reported noticeable and meaningful improvements in upper limb function following the AdaptRehab VR intervention. These perceived gains encompassed increased range of motion, improved muscle strength, enhanced coordination, greater fine motor control, reduced pain or discomfort, and better performance in activities of daily living. Participants described these improvements as having a meaningful impact on their functional independence. For example, one participant noted a significant increase in range of motion, stating, *“Before, I couldn’t lift my arm to reach high shelves. Now I can.”* Others highlighted improvements in strength and hand function, commenting, *“My hand feels stronger, and I can grip things better than before.”* Enhanced movement quality and coordination were also frequently reported, as reflected by one participant who explained, *“The coordination exercises helped me use my hand more smoothly.”* Several participants further described improvements in fine motor tasks, such as grasping and manipulating small objects, which translated into greater ease in performing everyday activities. In addition, 15% of participants reported reduced pain or discomfort during movement, while others noted broader benefits, including improved mood, increased confidence, and greater motivation to participate in rehabilitation sessions.

**Theme 4: Comparison with traditional therapy:** When asked to compare their experiences, participants consistently rated the VR-based therapy more favorably than their previous experiences with traditional rehabilitation methods. The most prominent distinction was in the level of engagement and the perception of the activities themselves. One participant contrasted the novelty of the AdaptRehab VR system with the monotony of conventional exercises, stating, *“Traditional exercises felt repetitive. The games were always different.”* This directly influenced their enthusiasm for attending therapy, as another admitted, *“I actually looked forward to therapy with the VR. With regular therapy, I sometimes wanted to skip.”* Crucially, this increased engagement was linked to a belief in its superior efficacy; participants felt the enjoyable nature of the tasks led to more intensive practice, with one explaining, *“I feel like I improved faster with the games because I practiced more movements.”*

**Theme 5: Usability and technical experience:** The system demonstrated a high level of usability, with 61% of participants reporting they found it easy to understand and navigate independently from an early stage. While 39% indicated they required initial assistance to become familiar with the setup, this need decreased markedly over subsequent sessions, suggesting a gentle but manageable learning curve. Technical issues were minimal, reported by only 8% of the sample. Crucially, when these did occur, they were described as minor and were quickly resolved by the attending physiotherapists, ensuring that the technical challenges did not significantly detract from the overall therapeutic experience.

**Theme 6: Suggestion for improvement:** Participants offered several constructive suggestions for enhancing the AdaptRehab VR system, providing valuable insights for future development. The most frequently cited recommendation, mentioned by 67% of participants, was for greater game variety and incorporate culturally adapted, locally relevant exercises, with the rationale that *“more games would keep it fresh longer.”* Closely related to this was the call for adjustable difficulty levels, made by 44% of the sample, who felt the challenge was not always optimally calibrated, noting, *“Sometimes it was too easy, sometimes too hard - more levels in between would help.”* A desire for more social interaction was expressed by 28% of participants, who thought, *“It would be fun to play with or compete with other patients.”* Finally, 22% expressed a wish for the technology to be accessible outside the clinical setting, stating, *“I wish I could use this at home too.”*

#### 3.5.2 Physiotherapist focus group discussion.

Thematic analysis of FGD with six physiotherapists revealed six major themes. The key (sub) concepts are presented in Fig 7.

**Fig 7:**
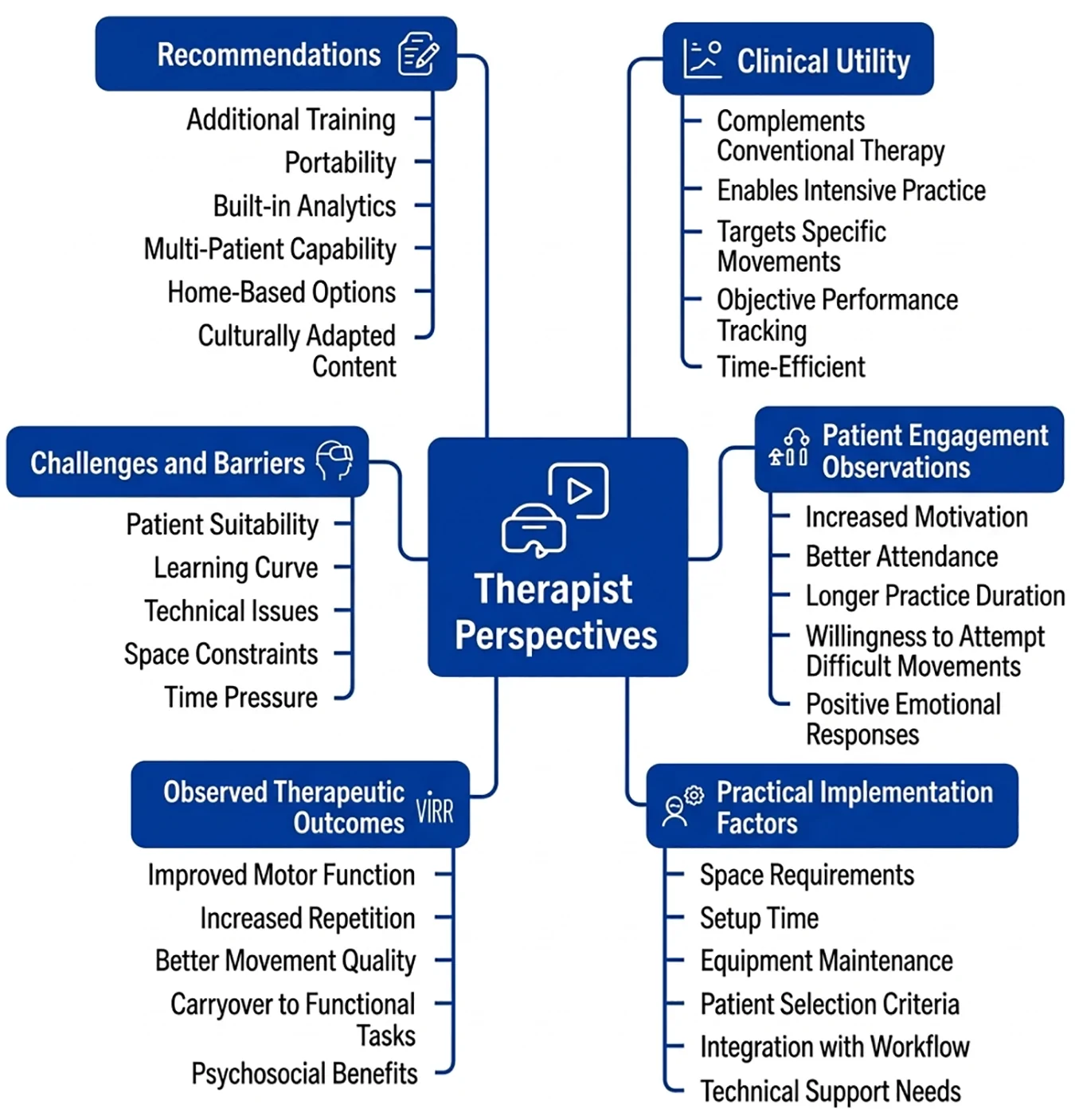
Key concepts of the thematic analysis for clinicians.

**Theme 1: Clinical utility and integration:** Physiotherapists reported that the AdaptRehab VR system integrated seamlessly into existing rehabilitation protocols, functioning as a valuable adjunct rather than a disruptive technology. They emphasized that the system complemented, rather than replaced, conventional therapy, with one physiotherapist stating, *“It complemented our conventional therapy nicely; we used it as an adjunct, not a replacement.”* A key advantage was its ability to complement therapist-led rehabilitation by enabling more intensive and repetitive upper limb practice than could be achieved through one-to-one therapy alone. As one participant explained, *“The system allowed us to provide more intensive upper limb practice than we could with one-on-one therapy alone.”* Physiotherapists also valued the flexibility of the system, which allowed them to tailor interventions by adjusting game parameters to target specific movement patterns and individual rehabilitation goals. In addition, the system provided objective performance tracking, enabling therapists to monitor patient progress over time and make evidence-informed adjustments to treatment. Several physiotherapists further noted that the system improved time efficiency by allowing patients to engage in structured, task-specific practice while therapists supervised and managed multiple aspects of rehabilitation.

**Theme 2: Patient engagement and motivation:** Physiotherapists consistently observed a marked increase in patient engagement and motivation when using the AdaptRehab VR system compared to conventional therapy. They noted a significant shift in attitude, particularly among patients who were typically difficult to engage. One physiotherapist stated, *“Patients who were previously reluctant to do their exercises became eager to participate.”* The inherent motivational features of the games, such as scoring and personal achievement, were identified as key drivers of this sustained interest. The competitive aspect, even when directed inwardly, proved powerful: *“The competitive element even competing against their own scores - kept them coming back.”* Perhaps most clinically significant was the observation that the immersive nature of the sysetem encouraged patients to perform movements they would otherwise avoid, with one physiotherapist reporting, *“We saw patients practicing movements in the system that they avoided in conventional therapy.”*

**Theme 3: Practical implementation considerations:** While the overall reception was positive, physiotherapists identified several practical factors that influence the successful implementation of the AdaptRehab VR system in a clinical setting. Logistical challenges were foremost among these considerations, particularly regarding the physical environment: *“We needed dedicated space, which can be challenging in a busy clinic.”* The time required for preparation was also noted as a factor, though it was seen as manageable, with one participant observing, *“Initial setup took about 10 minutes, which decreased with experience.”* Patient selection was another important consideration, as the technology was not deemed suitable for everyone; physiotherapists noted that *“some patients with cognitive impairments or severe visual problems weren’t suitable.”* Finally, the availability of technical support was highlighted as a facilitator for smooth operation: *“Having someone available for technical issues was helpful, though problems were rare.”*

**Theme 4: Observed therapeutic outcomes:** Beyond engagement, physiotherapists reported tangible improvements in both clinical measures and patient wellbeing. These outcomes reinforced the therapeutic value of the AdaptRehab VR system. One physiotherapist noted, *“We saw measurable improvements in motor function. The ARAT and BBT scores prove it, but we also saw it clinically.”* This was attributed in part to the high number of repetitions achievable, with a participant stating, *“Patients performed hundreds of repetitions per session. That’s far more than conventional therapy achieves.”* The quality of movement also saw positive changes; as one physiotherapist observed, “*Movement quality improved. Reaches became smoother, more coordinated. Less of the jerky, compensatory movements we often see.”* Crucially, this led to functional carryover, with patients reporting they *“were able to do things at home that they couldn’t before.”* The psychosocial benefits were equally striking. Physiotherapists noted *“improved mood, confidence, motivation,”* adding that *“some patients who were withdrawn became more engaged in all therapy.”* One participant encapsulated the holistic impact by stating, “*The sense of achievement was palpable. Patients would high-five us when they beat a high score. That emotional boost is therapeutic in itself.”*

**Theme 5: Challenges and barriers:** Physiotherapists also discussed specific challenges encountered during the system’s use, providing a balanced view of its application. Patient suitability remained a primary consideration, requiring careful assessment as *“not all patients are suitable. Those with severe cognitive impairment, uncorrected vision problems, or significant balance issues need careful assessment.”* While most patients adapted well, some faced a learning curve: *“There was a learning curve for some patients, especially older adults with no technology experience. But most picked it up within 2-3 sessions.”* Logistical constraints, such as space and time, were recurring themes. One participant reiterated that *“space was our biggest practical constraint. A dedicated AdaptRehab VR system room would be ideal, but we made it work with a partitioned area.”* Technical issues were noted but described as infrequent and manageable: *“Occasional technical glitches - game freezing, controller disconnection -but these were rare and easily fixed.”* Finally, clinical workflows posed a challenge, as *“time pressure in a busy clinic sometimes made it hard to offer AdaptRehab VR to everyone who wanted it. We had to prioritize.”* This was compounded by patient demand, with some individuals desiring more than the study protocol allowed.

**Theme 6: Recommendations for future implementation:** The physiotherapists offered several forward-looking recommendations to enhance the system’s utility and facilitate its broader adoption in clinical practice. The need for comprehensive training was a primary suggestion, with one physiotherapist advocating for *“more training on troubleshooting and optimizing exercise selection for different impairments”* to maximize therapeutic effectiveness. Improving the physical design of the equipment was also a key theme, as greater *“portability would allow us to serve patients in different areas”* of a facility. To support clinical decision-making and demonstrate efficacy, physiotherapists expressed a strong desire for enhanced data capabilities, such as *“built-in analytics showing patient progress over time.”* Finally, to increase efficiency and potentially introduce a new social dynamic to therapy, participants suggested exploring “multi-player options,” envisioning a future where *“group sessions with multiple patients using AdaptRehab VR system simultaneously could increase efficiency.”*

## 4. Discussion

This study investigated the feasibility, acceptability, and preliminary effectiveness of the AdaptRehab VR system for upper limb rehabilitation among stroke survivors in resources-limited environment. The findings demonstrate that the system is highly feasible, well-tolerated, and associated with significant functional improvements, supporting its potential as a valuable rehabilitation tool in LMIC settings.

The predefined feasibility criteria were achieved, with a retention rate of 90%, adherence of 93%, acceptable usability (SUS: 76.2 ± 12.1), and no serious intervention-related adverse events. These findings support the feasibility of progressing to a larger controlled trial, thus, further evaluation using a randomized controlled design is required to establish the intervention’s effectiveness [59,66]. The high adherence rate further demonstrates the feasibility and acceptability of delivering AdaptRehab VR system-based rehabilitation in this context. These rates compare favorably with previous feasibility studies of imVR rehabilitation in HIC settings [67,68] and are particularly noteworthy given the resource-constrained environment and participants’ limited prior technology exposure.

The SUS score of 76.2 ± 12.1 indicates good usability, exceeding the acceptable threshold of 68 [61]. This finding is encouraging, suggesting that stroke survivors with varying educational backgrounds and technology experience can successfully interact with the system. The particularly high ratings for ease of use (Q3: 4.61 ± 0.61) and confidence (Q9: 4.50 ± 0.71) suggest that the system’s design effectively accommodated users with diverse abilities.

The UEQ results further support the system’s positive reception, with all scales exceeding the positive evaluation threshold of 0.8. The attractiveness (1.92 ± 0.84) and novelty (1.86 ± 0.79) scales reaching “Excellent” benchmark levels indicate that participants found the system engaging and appealing. These findings align with previous research demonstrating that gamified rehabilitation approaches enhance motivation and engagement [25,26,69].

The minimal SSQ scores demonstrate excellent safety and tolerability of the system. The absence of moderate or severe symptoms and the low prevalence of even mild symptoms (primarily sweating and fatigue) suggest that the system is appropriate for stroke survivors, including those in the subacute and chronic stages. These findings contrast with earlier concerns about cybersickness in imVR rehabilitation [70,71] and may reflect the system’s adaptive design, gradual exposure protocol, and the short session durations (30 minutes) employed.

The significant improvements across functional outcome measures are encouraging, though must be interpreted cautiously given the uncontrolled, pre-post design. Although minimal clinically important differences (MCIDs) were not predefined in this feasibility study, the observed improvements provide preliminary evidence of potential clinical relevance. The ARAT improvements (15.6 points) exceeded the MCID of 5–6 points in chronic stroke survivors [72], and BBT improvements (7 blocks) also exceeded the MCID of 5–6 blocks [73]. These improvements likely reflect multiple mechanisms: the intensive, repetitive practice facilitated by the exercises [29,30]; enhanced motivation leading to greater effort and engagement [25,26]; and the task-specific training targeting functional movements [74]. Importantly, participants received the AdaptRehab VR system intervention as an adjunct to conventional therapy, suggesting that AdaptRehab VR system can effectively supplement rather than replace traditional approaches; however, given the feasibility design, small sample size, and absence of a control group, the effect estimates should be interpreted as preliminary and require confirmation through a larger randomized controlled trial.

Although participants demonstrated improvements following the AdaptRehab VR intervention, the single-arm pre–post design precludes causal inferences regarding intervention effectiveness. The observed changes may have been influenced by natural recovery, repeated exercises, participant adaptation, or other uncontrolled factors. Consequently, these findings should be interpreted as preliminary evidence of feasibility and potential clinical benefit rather than definitive evidence of efficacy. A fully powered randomized controlled trial with an appropriate comparison group is warranted to establish the efficacy of AdaptRehab VR system for upper limb stroke rehabilitation.

In addition, pre-intervention cognitive screening was not performed, which may have limited the identification of participants with cognitive difficulties affecting engagement. One participant (P20) discontinued due to cognitive impairments that limited their ability to understand instructions and interact with the AdaptRehab VR intervention. Future studies will incorporate standardized cognitive screening (Montreal Cognitive Assessment (MoCA) or the Mini-Mental State Examination (MMSE)) before enrolment.

The qualitative findings also provide rich context for understanding the quantitative results. The strong preference for AdaptRehab VR-based exercises over traditional therapy, expressed by most participants, aligns with the high SUS and UEQ scores. The motivational features identified, audio-visual feedback, scoring, progression, and immersion are consistent with self-determination theory [75], suggesting that the system supports autonomy, competence, and relatedness needs.

Physiotherapist perspectives highlighted the clinical utility of the system, particularly its ability to provide intensive practice while freeing physiotherapists to attend to other patients. This efficiency gain is particularly relevant in LMIC settings where physiotherapist shortages are acute [15,16]. The identification of practical implementation considerations, space requirements, setup time, patient selection criteria, provides valuable guidance for future scaling efforts.

The findings align with recent systematic review demonstrating the efficacy of imVR for upper limb stroke rehabilitation [31]. However, this study extends previous work by demonstrating feasibility and effectiveness in an LMIC context, while most imVR research has been conducted in HIC. The effect sizes observed are comparable to or exceed those reported in meta-analyses of imVR rehabilitation [32,76], suggesting that the system’s culturally adapted design may enhance its effectiveness.

The successful implementation addresses several challenges identified in previous LMIC-focused research [21,33,35], including limited technical expertise, cultural sensitivity issues, and language barriers. By developing a system specifically for this context, with local input during design and incorporating culturally relevant exercise elements, we overcame many barriers that have hindered technology adoption in LMICs [45].

Several limitations should be acknowledged. First, the uncontrolled pre-post design limits causal inference regarding functional improvements; regression to the mean, natural recovery, and concurrent conventional therapy may have contributed to observed gains. Therefore, concurrent therapies may have influenced participants’ outcomes. Consequently, the observed improvements should be interpreted carefully, as the specific contribution of AdaptRehab VR system cannot be fully distinguished from the effects of other ongoing rehabilitation interventions

Second, the sample size, while adequate for feasibility objectives, precludes subgroup analyses examining differential effects by stroke characteristics, demographics, or technology experience. Third, the relatively short intervention period (3 weeks) does not address long-term retention or maintenance effects. Fourth, the single-site design limits generalizability to other Ethiopian or LMIC settings with different populations or resources. Fifth, the lack of blinding in assessments may have introduced bias, though objective outcome measures partially mitigate this concern.

Thus, the findings suggest that the AdaptRehab VR system intervention may have contributed to improvements in upper limb rehabilitation outcomes among the participants. However, these results should be interpreted with caution because of the non-randomized study design, the relatively small sample size, and the possibility of selection bias. Variations among participants and potential measurement inaccuracies may also have influenced the outcomes.Furthermore, the lack of random allocation and long-term follow-up limits the generalizability of the findings. Future studies using larger samples and randomized controlled designs are recommended to further evaluate and confirm these preliminary results.

Despite these limitations our findings have several implications. First, the demonstrated feasibility supports progression to a fully powered randomized controlled trial to establish definitive effectiveness. Such a trial should include longer follow-up periods, cost-effectiveness analysis, and exploration of optimal dosing parameters. Second, the high acceptability suggests that AdaptRehab VR could be a valuable tool for addressing rehabilitation access gaps in LMICs. The system’s ability to provide intensive, engaging therapy with minimal physiotherapist supervision could help extend rehabilitation services to underserved populations [17]. Future implementation research should explore models for scaling, including community-based and potentially home-based applications. Third, the qualitative findings regarding motivational features and implementation considerations provide guidance for system refinement. Participant suggestions for increased game variety, adjustable difficulty, and social features should inform future development iterations.

Overall, the results suggest that the AdaptRehab VR system is a promising and practical option for upper limb stroke rehabilitation. It supports key principles of recovery such as active practice, feedback, and motivation, which are well established in rehabilitation theory. The findings are in line with previous studies on imVR in stroke rehabilitation. However, since this was a small feasibility study, the results should be interpreted with caution. Larger controlled trials are needed to confirm these effects.

## 5. Conclusions

This study successfully demonstrates that the AdaptRehab VR system is a feasible, acceptable, and safe intervention for upper limb stroke rehabilitation in a resource-limited Ethiopian setting. The findings bridge a critical gap in the literature by showing that an immersive virtual reality system, co-designed with local stakeholders and incorporating culturally relevant elements, can be effectively delivered despite the infrastructural and workforce constraints typical of low- and middle-income countries.

The positive reception from both stroke survivors and physiotherapists, combined with the observed clinical improvements, supports the system’s potential as a valuable adjunct to conventional rehabilitation. Importantly, the intervention was well-tolerated, with no serious adverse events, reinforcing its suitability for this population.

Looking ahead, several key steps are required to translate these promising feasibility findings into widespread clinical practice. First, a fully powered randomized controlled trial is essential to establish definitive evidence of clinical effectiveness, incorporating longer follow-up periods and blinded assessments to confirm the durability of functional gains. Second, implementation science research is needed to address the practical barriers identified in this study—particularly space constraints, patient selection criteria (including cognitive screening), and integration into existing clinical workflows. Third, cost-effectiveness analyses are critical to inform policy and funding decisions in resource-constrained health systems. Fourth, future development should explore home-based delivery models to overcome transportation barriers and extend access to rural populations. Finally, ongoing cultural adaptation and language expansion will be vital to ensure the system remains relevant and acceptable across Ethiopia’s diverse communities.

If these research priorities are addressed, the AdaptRehab VR system has the potential to meaningfully reduce the disability burden of stroke in Ethiopia and similar contexts, ultimately improving functional independence, community participation, and quality of life for stroke survivors.

## Supporting information

S1 File System Usability Scale. Participant-level System Usability Scale (SUS) data collected during the study.
(XLSX)

S2 File Simulator Sickness Questionnaire. Participant-level Simulator Sickness Questionnaire (SSQ) data collected during the study.
(XLSX)

S3 File User Experience Questionnaire. Participant-level User Experience Questionnaire (UEQ) data collected during the study.
(XLSX)

S4 File Action Research Arm Test. Participant-level Action Research Arm Test (ARAT) data collected during the study.
(XLSX)

S5 File Box and Block Test. Participant-level Box and Block Test (BBT) data collected during the study.
(XLSX)

S6 File Inclusivity in Global Research Questionnaire. Completed Inclusivity in Global Research Questionnaire related to the study.
(PDF)

S7 File Study protocol. Original full-length study protocol submitted to the Ethics Committee for review and approval before commencement of the study.
(PDF)

S8 File TREND checklist. Completed TREND checklist showing the location of relevant reporting items in the manuscript.
(PDF)
