# Supplementary material for: Feasibility, acceptability, and preliminary efficacy of AdaptRehab VR, an immersive virtual reality system for upper limb stroke rehabilitation: A mixed-methods study in Ethiopia
Source: PLoS One. 2026 Oct 9;21(10):e0360216. doi: 10.1371/journal.pone.0360216 (PMC13652278; doi:10.1371/journal.pone.0360216)
Supplement: Supplementary file 2 [file pone.0360216.s007.pdf]

AdaptRehab VR: Exploring the Feasibility of Immersive Virtual Reality for Upper Limb Stroke Rehabilitation in Ethiopia

Chala Diriba Kenea [1,2], Teklu Gemechu Abessa [2,3], Dheeraj Lamba [4], Bruno Bonnechere [2,5,6]

1 Department of Information Science, Faculty of Computing and Informatics, Jimma Institute of Technology, Jimma University, Jimma P.O. Box 378, Oromia, Ethiopia

2 REVAL Rehabilitation Research Center, Faculty of Rehabilitation Sciences, Hasselt University, Diepen Beek, Belgium.

3 Department of Special Needs and Inclusive Education, Jimma University, Jimma, Ethiopia

4 Department of Physiotherapy, Faculty of Medical Sciences, Institute of Health, Jimma University, Jimma, Ethiopia.

5 Technology-Supported and Data-Driven Rehabilitation, Data Sciences Institute, Hasselt University, Diepen Beek, Belgium.

6 Department of PXL—Healthcare, PXL University of Applied Sciences and Arts, 3500 Hasselt, Belgium

Summary

Background: Stroke poses a significant risk for long-term disability on a global scale, particularly in low- and middle-income countries (LMICs). Impairment in upper limp function resulting from stroke can significantly hinder a person's ability to carry out daily activities and participate in community life. Conventional therapy methods have been employed, but they may require increased engagement and adherence. Immersive Virtual Reality (imVR) systems provide a promising avenue for rehabilitation to improve upper limb function, motivation, and accessibility. **Objective**: The study aims to evaluate the feasibility, acceptability, enjoyment, and safety of the AdaptRehab VR system in context of Ethiopia, offering valuable insights to inform the planning of a future randomized controlled trial.

Method: A mixed methods design approach will be used. The data will be included both quantitative and qualitative data. The quantitative includes upper extremity outcome measures, usability, user experience and cybersickness while qualitative will be the acceptability of the system using interview and focus group discussion. The proposed study's overall reporting and methodology adhere to CONSORT 2010 Extension for Pilot and Feasibility Studies (Non-Randomized). The study will be carried out in the physiotherapy clinic center of the Jimma University Teaching Hospital in Jimma, southwest Ethiopia. After reviewing their medical records and determining their eligibility, adults (age greater than 18) who present to the study setting with subacute and chronic, and those who consent to be contacted for research will be recruited. The therapists will collect the baseline data and post intervention data from the participants such upper extremity outcome measures, usability, user experience and cybersickness. Qualitative data will also be collected from all physiotherapists and nurses providing therapy services at the JUTH physiotherapy clinic to get their reflection on the proposed AdaptRehab VR system suitability and acceptability for stroke patients' upper limb rehabilitation. The sample size for this feasibility study is justified based on the estimated patient flow in the study context and the need to obtain sufficient data on the feasibility and acceptability of the intervention. The study plans to recruit 20 stroke patients over 3 weeks, considering a 15% non-participation rate and a 15% exclusion rate. Additionally, based on their consent, we will include all physiotherapists and trained nurses providing therapy services at the JUTH physiotherapy clinic.

Keywords: feasibility, use acceptance, immersive virtual reality, upper limb, rehabilitation, and stroke

Introduction

Stroke represents a significant global health burden, affecting fifteen million individuals annually and causing over 5.5 million deaths worldwide [1,2]. Notably, two-thirds of stroke-related mortality occurs in low- and middle-income countries (LMICs) [3]. The disparity is further emphasized by the concentration of over 87% of stroke-related disability-adjusted life years (DALYs) in LMICs, a rate approximately seven times higher than in high-income countries (HICs) [4]. This substantial burden underscores the critical importance of accessible rehabilitation services [5].

LMICs face considerable challenges in delivering equitable, high-quality rehabilitation care [6]. The most pressing concerns include a severe shortage of rehabilitation professionals, with some regions reporting as few as 0.5 therapists per 10,000 population, and inadequate infrastructure, particularly in rural areas [7].

In response to these challenges, various international initiatives have emerged. The World Health Organization's 2030 Rehabilitation Initiatives represents a significant global effort to address rehabilitation needs [8]. Regional priorities are also evolving, as evidenced by health-focused policies such as Africa's Agenda 2063 [9]. At the national level, countries like Ethiopia have integrated rehabilitation services into their healthcare frameworks, as demonstrated by the inclusion of rehabilitation in both the Health Sector Transformation Plan II and the five-year National Rehabilitation and Assistive Technology Strategic Plan [10].

With substantial effects on physical, cognitive, and psychosocial functioning, stroke is one of the main causes of long-term disability in all over the world [11]. Ethiopia had an age-standardized stroke incidence rate of 151.5 per 100,000, with ischemic stroke accounting for a large portion of incidents, according to the Global Burden of Disease research [12]. Stroke-related impairment and a decreased quality of life are major public health concerns in Ethiopia, where the condition is becoming more prevalent stroke-related impairment and reduced quality of life are major public health concerns in Ethiopia, where the condition is becoming more prevalent [13].

Fifty to eighty percent of stroke survivors experience upper-limb impairment [14]. This can have a major negative influence on a person's capacity to live freely and engage in community life by reducing their mobility, dexterity, and functional independence in everyday tasks [15]. Deficits in muscle strength, range of motion, coordination, and sensory processing are some of the characteristics of stroke-related upper limb impairment. These disabilities may limit an individual's ability to take care of themselves, participate

fully in social and community-related work, or engage in leisure activities leading to profound consequences [16,17]. Numerous stroke survivors fail to receive what they need to regain upper limb function in resource-constrained regions like Jimma, where access to comprehensive stroke rehabilitation services is scarce, and healthcare professionals are not enough [18].

Basic self-care activities that people conduct regularly are called Activities of regular Living (ADLs). feeding, bathing, grooming, dressing, functional mobility, using toilets, and washing oneself after passing urine are a few of them. Keeping one's independence and quality of living requires the effective completion of certain ADLs. For this task to be completed, the upper limb function is crucial [19, 20].

The recent development and democratization of technology has led to the development of technology-supported rehabilitation. New technology could be a potential solution to increase the provision of rehabilitation services despite the shortage of healthcare professionals, especially in LMICs. Amongst the different technologies, one of the most promising one, for stroke rehabilitation is Virtual Reality (VR).

imVR has been shown to be effective in rehabilitation by reducing pain, accelerating recovery times, providing realistic environments, decreasing dependence on rehabilitation personnel, enabling increased treatment intensity and frequency, facilitating creative treatment delivery, standardizing therapeutic activities, and improving overall recovery outcomes [21,22]. Key features include real-time feedback [23], customizable and adaptive environments [24], enhanced motivation [25,26], safe and controlled environments [27], and multisensory stimulation including visual, auditory, and haptic feedback [28]. Additionally, imVR facilitates essential repetitive exercises [29,30], with numerous studies demonstrating its effectiveness in stroke rehabilitation [31,32]. Furthermore, recent studies have demonstrated the feasibility of implementing advanced rehabilitation technologies in LMICs [33], suggesting potential for significant enhancement of rehabilitation services in regions facing professional shortages [33]. However, the implementation of imVR in healthcare, particularly for rehabilitation in LMICs, faces multiple significant challenges. These include limited technical expertise for development and maintenance [21], insufficient multidisciplinary collaboration [33], inadequate government funding [34], and shortage of rehabilitation services and technologies [35-41]. Furthermore, existing imVR applications, primarily designed for HICs, often fail to address LMIC-specific needs and local technical realities.

The adaptation of HICs developed imVR applications in LMICs is hindered by cultural sensitivity issues, proprietary restrictions, and frequent technological updates. These challenges are compounded by inadequate training and awareness of imVR technologies [41], limited research on effectiveness in LMIC contexts [42], and significant cultural and language barriers, as most systems are designed in English [43].

Despite the increasing stroke patient population in LMICs, there are currently no commercially available imVR solutions incorporating local cultural factors [44]. This gap is particularly critical given that 50-80% of stroke survivors experience upper limb impairment [45], significantly impacting their activities of daily living (ADLs) and contributing to perceived disability. This integrates concepts from imVR, and motor learning, all of which may enhance the efficacy and delivery of rehabilitation therapies [46, 47]. Evaluating the potential use of the AdaptRehab VR system for upper limb rehabilitation among stroke patients in Jimma, Southwest Ethiopia, is the goal of this feasibility study. The AdaptRehab VR system includes six games such as Basket Bloom, Strike Zone, TapQuest, FruitFall Frenzy, and Precision Pitch each designed to target different therapeutic goals. This study is important as there is limited prior research on the specific intervention technique used and to consider the sociocultural health beliefs of the target population. The lack of data on the intervention in the particular context makes this study a significant contribution to the field. The study will offer important insights to guide the design and execution of a larger-scale randomized controlled trial by evaluating the feasibility, acceptability, enjoyment, and safety of this intervention [48]. The primary goals are to investigate the feasibility and acceptability of the proposed AdaptRehab VR system for ULSR. The assessment of the intervention's secondary outcomes will be evaluated.

Methodology

Design

A mixed methods design approach will be used. The data will be included both quantitative and qualitative data. The quantitative includes upper extremity outcome measures, usability, user experience and cybersickness which will be collected from participants while qualitative will be the acceptability of the system using interview from participants and focus group discussion from physiotherapists. The proposed study's overall reporting and methodology adhere to CONSORT 2010 Extension for Pilot and Feasibility Studies (Non-Randomized) [49-51].

Setting

The study will be carried out in the physiotherapy center of the Jimma University Teaching Hospital (JUTH) in Jimma, southwest Ethiopia.

Participants

The participants will be the patients who follow their rehabilitation program at JUTH. After reviewing their medical records and determining their eligibility, adults (age greater than 18) who present to the

study setting with subacute and chronic [54], and those who consent to be contacted for research will be recruited. The therapists will rate their satisfaction with the intervention sessions and record any unfavorable events to participate.

Qualitative data will also be collected from all physiotherapists and nurses providing therapy services at the JUTH physiotherapy center to get their reflection on the proposed AdaptRehab VR system suitability and acceptability for stroke patients' upper limb rehabilitation.

Sample size

The sample size for this feasibility study is justified based on the estimated patient flow in the study context and the need to obtain sufficient data on the feasibility and acceptability of the intervention. The study plans to recruit 20 stroke patients over 3 weeks, considering a 15% non-participation rate and a 15% exclusion rate. This sample size is expected to provide adequate data, with the confidence interval width $[1.96 \times \sqrt{(p \times (1-p) / n)}]$ for an estimated 80% drop-out rate being within ±15.56% at a 95% confidence level. Additionally, based on their consent, we will include all physiotherapists and trained nurses providing therapy services at the JUTH physiotherapy clinic [55].

Eligibility criteria

Eligibility Criteria

Inclusion criteria were adults aged 18 years or older with a diagnosis of ischemic or hemorrhagic stroke, with stroke onset of six months or more prior to enrollment, who were in the subacute or chronic stage of recovery and presented with upper limb impairment. Participants were required to be medically stable, able to understand and follow instructions, and to have adequate cognitive and visual abilities to safely interact with the AdaptRehab VR system. **Exclusion criteria** included a diagnosis of severe cognitive impairment, significant visual impairment that interfered with VR interaction, the presence of severe psychological or psychiatric disorders, and any medical condition that could compromise safe participation in VR-based rehabilitation.

Intervention

We will use the proposed AdaptRehab VR system to rehabilitate the upper limb. With an emphasis on enhancing upper limb function, activity of daily living, and quality of life, the proposed AdaptRehab VR system activities will be customized to each participant's unique requirements and treatment plan. Over

the course of the intervention, participants will get direction and assistance from a qualified physiotherapist, and the proposed AdaptRehab VR system will be set up in a specific area within the JUTH physiotherapy clinic. As appropriate, participants will be encouraged to practice the proposed AdaptRehab VR system independently in between supervised sessions.

Outcome Measures

This study's primary outcomes will be:

1. **Feasibility**: Recruitment rates (number of participants that we will approach, exclude, or agree for further follow-up), retention rate (number of participants who will withdraw, lost to follow-up or who will provide data), and adherence rate (number of individual who able to perform intervention as guided which is calculated from the ratio of the number of sessions performed (SP) and the prescribed number of sessions (PS)) to the intervention.
2. **System Usability Scale (SUS):** The SUS will be conducted at the end of the intervention, after the 9 sessions, by the therapists from the participants. The SUS consists of 10 questions with 5 response options based on a Likert scale. This will be conducted at the end of the intervention.
3. **User experience questionnaire (UEQ):** The UEQ uses a 7-point scale ranging from 1 to 7, for example, from unattractive to attractive. On this scale, [1] represents extremely negative / completely unattractive, [2] represents very negative / mostly unattractive, [3] represents moderately negative / somewhat unattractive,[4] represents neutral / neither attractive nor unattractive, [5] represents moderately positive / somewhat attractive, [6] represents very positive / mostly attractive, and [7] represents extremely positive / completely attractive. The UEQ consists of 26 questions and will be completed by the therapists from the participants at the end of the intervention. This will be conducted at the end of the intervention.
4. **Simulator Sickness Questionnaire:** The Simulator Sickness Questionnaire also conducted at the end of intervention. The data will be collected from participants by the therapists. The therapists rate how much each listed symptoms such as general discomfort, fatigue, headache and eyestrain are affecting them at that moment using a 4-point scale: 0 = not at all, 1 = mild, 2 = moderate and 3 = severe. This will be conducted at the end of the intervention.
5. **Acceptability**: participant willingness to employ the intervention in the future, participant happiness with the proposed AdaptRehab VR system, and participant perception of its usefulness. It will be measured by the system usability scale, customer satisfaction score, net promotor score, and also by focus group discussion from the therapist and in-depth interview with the patient.

Secondary outcome

Action Research Arm Test (ARAT): ARAT is another commonly used tool for assessing upper extremity function. It consists of four subtests: Grasp**,** Grip**,** Pinch**,** and Gross Movement, with the following score ranges: Grasp (0–18), Grip (0–12), Pinch (0–18), and Gross Movement (0–9). A total score is calculated

by summing the scores of all subdomains (range: 0–57), with higher scores indicating better hand dexterity. The assessments will be conducted two times: at baseline (pre-intervention) and at the end of the intervention (post-intervention).

Box and Block Test (BBT): The BBT is a widely used assessment of manual dexterity. It measures how many blocks a participant can transfer, one at a time, from one compartment of a box to another within 60 seconds. A higher number of blocks transferred indicate better hand and arm dexterity. The test is simple, quick to administer, and is commonly used in studies evaluating upper extremity function in stroke and rehabilitation settings. The assessments will be conducted two times: at baseline (pre-intervention) and at the end of the intervention (post-intervention). Other measurements could be clinical and demographic characteristics.

Data collection processes

Before the actual data collection, training will be given to the physiotherapist (three) who will be involved in the data collection and supervision of proposed AdaptRehab VR system and the protocol details for two days at JUTH. All quantitative data will be collected from patients; qualitative data will be collected from both patients and therapists. The data on sociodemographic, clinical, feasibility, , usability, user experience and cybersickness, acceptability, and secondary outcomes (upper extremity outcome measures) will be collected.

Data analysis and interpretation

Quantitative analysis

Descriptive statistics (frequencies, percentages, medians, and interquartile ranges) will present data on stroke patient's sociodemographic and clinical characteristics and outcomes data. Mean differences will be calculated to evaluate changes in daily living activities and quality of life and to estimate the sample size for future RCTs. Data will be entered into Epi data version 3.1 and then exported to SPSS 29.0 for analysis.

Qualitative analysis and interpretation

Thematic analysis will be used for qualitative data from in-depth interviews and focus group discussions (FGD). In-depth interviews and discussions will be audio-recorded, transcribed, and analyzed using framework analysis involving transcription, coding, developing a working thematic framework, data charting into the framework, and interpretation of data. Then, carefully review the transcripts to identify recurring themes and patterns within the data. This involves systematically coding the textual information

to categorize and organize the key ideas and perspectives expressed by participants. The coded data is then further explored to detect key themes that capture the core of the participant's experiences. And also we look for divergent or contradictory themes to gain a more refined understanding of the topic. MAX QDA software will be used for easy of this qualitative data analysis.

Data synthesis

We will use a parallel-results convergent synthesis design, in which qualitative and quantitative data are analyzed and presented separately, with integration occurring in interpreting results in the discussion section.

Recruitment

Stroke patients presenting to the clinic for therapy will be screened for eligibility and enrolled.

Pre and Post-intervention

The physical therapist will conduct a face-to-face interview, administer a questionnaire, and conduct in-depth interviews with patients and therapists following their consent to participate. Meanwhile, data on sociodemographic (age, sex, educational status, occupation, income, and residence); clinical characteristics (time since stroke, stroke type, stroke stage, and others from patient medical records); and outcome (acceptability, feasibility, experience with game, safety, ADL, and QoL,) will be collected at baseline and end line.

Study Timeline

The study will be conducted from May 2025 to March 2026 and is expected to be conducted over 5 to 6 weeks.

Ethical considerations

Participants will be treated according to principles of justice, beneficence, and respect for humans as stated in the “Declaration of Helsinki,” following Good Clinical Practice and institutional and international guidelines (23). Hence, all patients who fulfill the inclusion criteria are fully eligible to join the study regardless of gender, ethnic background, or minority status. Following consent to participate, patients will be informed verbally and in writing that they may decline participation or leave the study at any time without negative influences. Deviations from the approved protocol will not be allowed except when necessary to protect participants. Deviations that can influence the study conduct, outcomes, safety of

participants, and procedures will require an amendment to the protocol. The Institutional Review Board at Jimma University will examine and approve the study protocol. The study's participants will be made aware of their ability to withdraw at any time and the voluntary nature of their involvement. Data regarding participants will remain confidential, and any adverse events will be quickly reported and handled.

Risk management

The intervention can be considered safe with serious adverse effects unlikely. Despite the safety of the intervention, we acknowledge the necessity of protecting patients by carefully monitoring them. Adverse events will be monitored by reports from patients. The therapist will address adverse events after each therapeutic session by clinicians using a standardized management protocol physiotherapy department. Investigators will meet weekly to monitor the study's progress. Following Ethiopian National Ethics Guidelines, events that may influence the safety of participants will be reported to the JUTH and ethics committee. No data will be generated by this protocol. All data will be collected during the proposed study and recorded and stored in a coded form where possible. Only the research team and the ethics committee can access participants' records unless the law mandates. Study results will be published in a peer-reviewed journal and presented at conferences. Investigators will ensure that all participants are not

References

1.Sun, H.; Ma, B.; Jin, C.; Li, Z.; Song, X.; Bu, Y.; Liu, T.; Han, X.; Yang, X.; Yang, K.; et al. Global, Regional, and National Burdens of Stroke in Children and Adolescents From 1990 to 2019: A Population-Based Study. *Stroke* **2024**, *55*, 1543–1553, doi:10.1161/STROKEAHA.123.044827.

2. Donkor, E.S. Stroke in the 2 1 s t Century: A Snapshot of the Burden, Epidemiology, and Quality of Life. *Stroke Res. Treat.* **2018**, *2018*, 1–10, doi:10.1155/2018/3238165.

3. El Hajj, M.; Abdo, R.; Assaf, S.; Lahoud, N. Stroke Management in Developing Countries. In *Handbook of Medical and Health Sciences in Developing Countries*; Al-Worafi, Y.M., Ed.; Springer International Publishing: Cham, 2023; pp. 1–31 ISBN 978-3-030-74786-2.

4. Johnston, S.C.; Mendis, S.; Mathers, C.D. Global Variation in Stroke Burden and Mortality: Estimates from Monitoring, Surveillance, and Modelling. *Lancet Neurol.* **2009**, *8*, 345–354.

5. Jesus, T.S.; Landry, M.D.; Dussault, G.; Fronteira, I. Human Resources for Health (and Rehabilitation): Six Rehab-Workforce Challenges for the Century. *Hum. Resour. Health* **2017**, *15*, 8, doi:10.1186/s12960-017-0182-7.

6. Europe, W.H.O.R.O. for Rehabilitation: Fact Sheet on Sustainable Development Goals (SDGs): Health Targets. **2019**.

7. Felter, C.E.; Zalewski, K.; Jermann, R.; Palmer, P.L.; Baier, A.E.; Falvey, J.R. Rural Health: The Dirt Road Less Traveled. *Phys. Ther.* **2022**, *102*, pzac112.

8. Rehabilitation 2030 Available online: https://www.who.int/initiatives/rehabilitation-2030 (accessed on 14 January 2025).

9. Goals & Priority Areas of Agenda 2063 | African Union Available online: https://au.int/agenda2063/goals (accessed on 14 January 2025).

10. Ethiopia Health Sector Transformation Plan (2020/21-2024/25) | Global Financing Facility Available online: https://www.globalfinancingfacility.org/resource/ethiopia-health-sector-transformation-plan-202021-202425 (accessed on 14 January 2025).
11. Babkair LA, Chyun D, Dickson VV, Almekhlafi MA. The Effect of Psychosocial Factors and Functional Independence on Poststroke Depressive Symptoms: A Cross-Sectional Study. J Nurs Res. 2022 Feb;30(1):e189.
12. GBD 2019 Stroke Collaborators. Global, regional, and national burden of stroke and its risk factors, 1990-2019: a systematic analysis for the Global Burden of Disease Study 2019. Lancet Neurol. 2021 Oct;20(10):795–820.
13. Misganaw A, Naghavi M, Walker A, Mirkuzie AH, Giref AZ, Berheto TM, et al. Progress in health among regions of Ethiopia, 1990–2019: a subnational country analysis for the Global Burden of Disease Study 2019. The Lancet. 2022 Apr 2;399(10332):1322–35.
14. Rafsten L, Meirelles C, Danielsson A, Sunnerhagen KS. Impaired Motor Function in the Affected Arm Predicts Impaired Postural Balance After Stroke: A Cross Sectional Study. Front Neurol [Internet]. 2019 Aug 21 [cited 2024 Jun 12];10. Available from: https://www.frontiersin.org/journals/neurology/articles/10.3389/fneur.2019.00912/full
15. Gao J, Gao Q, Huo L, Yang J. Impaired Activity of Daily Living Status of the Older Adults and Its Influencing Factors: A Cross-Sectional Study. Int J Environ Res Public Health. 2022 Nov 24;19(23):15607.
16. Roby-Brami A, Jarrassé N, Parry R. Impairment and Compensation in Dexterous Upper-Limb Function After Stroke. From the Direct Consequences of Pyramidal Tract Lesions to Behavioral Involvement of Both Upper-Limbs in Daily Activities. Front Hum Neurosci [Internet]. 2021 [cited 2024 Jun 12];15. Available from: https://www.ncbi.nlm.nih.gov/pmc/articles/PMC8255798/
17. Tomita Y, Rodrigues MRM, Levin MF. Upper Limb Coordination in Individuals With Stroke: Poorly Defined and Poorly Quantified. Neurorehabil Neural Repair. 2017 Oct 1;31(10–11):885–97.
18. Kayola G, Mataa MM, Asukile M, Chishimba L, Chomba M, Mortel D, et al. Stroke Rehabilitation in Low- and Middle-Income Countries: Challenges and Opportunities. Am J Phys Med Rehabil. 2023 Feb 1;102(2):S24–32.
19. Aderinto N, Olatunji G, Abdulbasit MO, Edun M, Aboderin G, Egbunu E. Exploring the efficacy of virtual reality-based rehabilitation in stroke: a narrative review of current evidence. Ann Med. 55(2):2285907.
20. Li Y, Wang Q, Liu XL, Hui R, Zhang YP. Effect of the physical rehabilitation program based on self-care ability in patients with acute ischemic stroke: a quasi-experimental study. Front Neurol [Internet]. 2023 Jun 9 [cited 2024 Jul 13];14. Available from: https://www.frontiersin.org/journals/neurology/articles/10.3389/fneur.2023.1181651/full
21 Antonopoulos, P.; Fokides, E.; Koutromanos, G. Understanding Learning and Learning Experience in Immersive Virtual Reality. Technol. Knowl. Learn. 2024, 1–30.
22. Rose, T.; Nam, C.S.; Chen, K.B. Immersion of Virtual Reality for Rehabilitation-Review. Appl. Ergon. 2018, 69, 153–161.
23. Dias, P.; Silva, R.; Amorim, P.; Lains, J.; Roque, E.; Serôdio, I.; Pereira, F.; Santos, B.S. Using Virtual Reality to Increase Motivation in Poststroke Rehabilitation. IEEE Comput. Graph. Appl. 2019, 39, 64–70.
24. Kern, F.; Winter, C.; Gall, D.; Käthner, I.; Pauli, P.; Latoschik, M.E. Immersive Virtual Reality and Gamification within Procedurally Generated Environments to Increase Motivation during Gait Rehabilitation. In Proceedings of the 2019 IEEE Conference on Virtual Reality and 3D User Interfaces (VR); IEEE, 2019; pp. 500–509.
25. Haghedooren, E.; Haghedooren, R.; Langer, D.; Gosselink, R. Feasibility and Safety of Interactive Virtual Reality Upper Limb Rehabilitation in Patients with Prolonged Critical Illness. Aust. Crit. Care 2024, 37, 949–956.
26. Bektic, M. AN EVALUATION OF VR GAMES AND HAPTIC FEEDBACK FOR UPPER LIMB REHABILITATION OF PARKINSON'S PATIENTS. Master's Thesis, Kent State University, 2024.
27. Pacchierotti, C.; Chinello, F.; Koumaditis, K.; Di Luca, M.; Ofek, E.; Georgiou, O. Haptics in the Metaverse: Haptic Feedback for Virtual, Augmented, Mixed, and eXtended Realities. IEEE Trans. Haptics ToH 2024.

28. Castillo, J.F.V.; Muñoz, J.E.; Lopez, D.; Lopez, J.F.; Gallo, O.H. Exploratory Analysis of Game Metrics of a Multi-Session Study of a Virtual Reality Exergame for Stroke Rehabilitation. In Proceedings of the 2024 IEEE 12th International Conference on Serious Games and Applications for Health (SeGAH); IEEE, 2024; pp. 1–8.
29. Rodríguez-Fuentes, G.; Ferreiro-Gómez, E.; Campo-Prieto, P.; Cancela-Carral, J.M. Exergames and Immersive Virtual Reality as a Novel Therapy Approach in Multiple Sclerosis: Randomised Feasibility Study. J. Clin. Med. 2024, 13, 5845.
30. Kiper, P.; Godart, N.; Cavalier, M.; Berard, C.; Cieślik, B.; Federico, S.; Kiper, A.; Pellicciari, L.; Meroni, R. Effects of Immersive Virtual Reality on Upper-Extremity Stroke Rehabilitation: A Systematic Review with Meta-Analysis. J. Clin. Med. 2023, 13, 146.
31. Kenea, C.D.; Abessa, T.G.; Lamba, D.; Bonnechère, B. Immersive Virtual Reality in Stroke Rehabilitation: A Systematic Review and Meta-Analysis of Its Efficacy in Upper Limb Recovery. J. Clin. Med. 2025, 14, 1783, doi:10.3390/jcm14061783.
32. Boma, P.M.; Panda, J.; Ngoy Mande, J.P.; Bonnechère, B. Rehabilitation: A Key Service, yet Highly Underused, in the Management of Young Patients with Sickle Cell Disease after Stroke in DR of Congo. Front. Neurol. 2023, 14, 1104101, doi:10.3389/fneur.2023.1104101.
33. Kamalakannan, S.; Menon, B.; Solomon, J.M.; Musa, K.I. Editorial: Evidence on Low-Cost Technologies for Neurological Rehabilitation in Low and Middle-Income Countries. Front. Neurol. 2023, 14, doi:10.3389/fneur.2023.1323808.
34. Tamrat, E.G.; Gufue, Z.H.; Getachew, S.; Yifru, Y.M.; Gizaw, M. Factors Associated with the Longer-Term Unmet Supportive Care Needs of Stroke Survivors in Ethiopia: A Multicentre Cross-Sectional Study. BMJ Open 2022, 12, e053579.
35. Ayele, Z.A.; Molla, S.; Ahmed, A.; Worku, T.; Bezabih, A.S.; Mamo, B.T. Poor Treatment Outcomes and Associated Factors among Hospitalized Patients with Stroke at Hiwot Fana Comprehensive Specialized Hospital, Eastern Ethiopia. Front. Stroke 2023, 2, 1304664.
36. Melese, A.Z.; Weldetsadik, F.K.; Yalew, E.S.; Belay, G.G.; Gobezie, M.; Erikiu, G.A.; Fantanew, M.; Takele, M.D.; Yitayal, M.M. Post Stroke Fatigue and Associated Factors among Stroke Survivors at Tertiary Hospitals, Amhara Regional State, Ethiopia; Multicenter Cross-Sectional Study. 2024.
37. Sidelil, H.; Demissie, A.; Demissie, G.D.; Fikade, B.; Hailegebreal, S.; Tilahun, B. Attitude towards Tele Rehabilitation-Based Therapy Services and Its Associated Factors among Health Professional Working in Specialized Teaching Hospitals in Amhara Region, Northwest Ethiopia. Inform. Med. Unlocked 2023, 36, 101145.
38. Meseret, F. Discrepancy Between Rhetoric and Practice: A Qualitative Study Assessing Barriers Associated With Prisoner Rehabilitation at Hawassa Correctional Facility, Southern Ethiopia. Sage Open 2018, 8, 2158244017753866, doi:10.1177/2158244017753866.
39. Dendea, S.G.; Ababor, J.K.; Daba, K.T.; Molla, J.M. Perceived Barriers to Low Vision Rehabilitation Services among Eye Care Practitioners in Ethiopia: A Cross-Sectional Study. 2022.
40. Addis, D.; Mesele, H.A. Provision of Rehabilitation Services to People with Physical Impairment in Bahir Dar Physical Rehabilitation Center, North West Ethiopia.
41. Li, X.; Elnagar, D.; Song, G.; Ghannam, R. Advancing Medical Education Using Virtual and Augmented Reality in Low-and Middle-Income Countries: A Systematic and Critical Review. In Proceedings of the Virtual Worlds; MDPI, 2024; Vol. 3, pp. 384–403.
42. Diriba Kenea, C.; Gemechu Abessa, T.; Lamba, D.; Bonnechère, B. Technological Features of Immersive Virtual Reality Systems for Upper Limb Stroke Rehabilitation: A Systematic Review. Sensors 2024, 24, 3546.
43. Veras, M.; Labbé, D.R.; Furlano, J.; Zakus, D.; Rutherford, D.; Pendergast, B.; Kairy, D. A Framework for Equitable Virtual Rehabilitation in the Metaverse Era: Challenges and Opportunities. Front. Rehabil. Sci. 2023, 4, doi:10.3389/fresc.2023.1241020.

44. Abaza, H.; Marschollek, M. mHealth Application Areas and Technology Combinations: A Comparison of Literature from High and Low/Middle Income Countries. Methods Inf. Med. 2017, 56, e105–e122, doi:10.3414/ME17-05-0003.
45. Phelan, I.; Carrion-Plaza, A.; Furness, P.J.; Dimitri, P. Home-Based Immersive Virtual Reality Physical Rehabilitation in Paediatric Patients for Upper Limb Motor Impairment: A Feasibility Study. Virtual Real. 2023, 27, 3505–3520, doi:10.1007/s10055-023-00747-6.
46. Kushnir A, Kachmar O, Bonnechère B. STASISM: A Versatile Serious Gaming Multi-Sensor Platform for Personalized Telerehabilitation and Telemonitoring. Sensors. 2024 Jan;24(2):351.
47. Bowen DJ, Kreuter M, Spring B, Cofta-Woerpel L, Linnan L, Weiner D, et al. How We Design Feasibility Studies. Am J Prev Med. 2009 May;36(5):452–7.
48. CONSORT 2010 statement: extension to randomised pilot and feasibility trials | EQUATOR Network [Internet]. [cited 2024 Jul 10]. Available from: https://www.equator-network.org/reporting-guidelines/consort-2010-statement-extension-to-randomised-pilot-and-feasibility-trials/
49. Lancaster GA, Thabane L. Guidelines for reporting non-randomised pilot and feasibility studies. Pilot Feasibility Stud. 2019 Dec;5(1):114, s40814-019-0499–1.
50. Von Elm E, Altman DG, Egger M, Pocock SJ, Gøtzsche PC, Vandenbroucke JP. The Strengthening the Reporting of Observational Studies in Epidemiology (STROBE) statement: guidelines for reporting observational studies. The lancet. 2007;370(9596):1453–7.
51. Alvarez G, Cerritelli F, Urrutia G. Using the template for intervention description and replication (TIDieR) as a tool for improving the design and reporting of manual therapy interventions. Man Ther. 2016;24:85–9.
52. Shaw RB, Sweet SN, McBride CB, Adair WK, Martin Ginis KA. Operationalizing the reach, effectiveness, adoption, implementation, maintenance (RE-AIM) framework to evaluate the collective impact of autonomous community programs that promote health and well-being. BMC Public Health. 2019 Jun 24;19(1):803.
53. Hijikata N, Kawakami M, Ishii R, Tsuzuki K, Nakamura T, Okuyama K, et al. Item Difficulty of Fugl-Meyer Assessment for Upper Extremity in Persons With Chronic Stroke With Moderate-to-Severe Upper Limb Impairment. Front Neurol. 2020 Nov 16;11:577855.
54. RDS London [Internet]. [cited 2024 Jul 30]. Justify sample size for a feasibility study. Available from: https://www.rds-london.nihr.ac.uk/resources/justify-sample-size-for-a-feasibility-study/
55. Al-Durra M, Nolan RP, Seto E, Cafazzo JA. Prospective registration and reporting of trial number in randomised clinical trials: global cross sectional study of the adoption of ICMJE and Declaration of Helsinki recommendations. bmj [Internet]. 2020 [cited 2024 Jun 26];369. Available from: https://www.bmj.com/content/369/bmj.m982.abstract
