# Supplementary material for: Feasibility, acceptability, and preliminary efficacy of AdaptRehab VR, an immersive virtual reality system for upper limb stroke rehabilitation: A mixed-methods study in Ethiopia
Source: PLoS One. 2026 Oct 9;21(10):e0360216. doi: 10.1371/journal.pone.0360216 (PMC13652278; doi:10.1371/journal.pone.0360216)
Supplement: Supplementary file 4 [file pone.0360216.s009.docx]

# **Response to reviewers**

## **Reviewer #1:**

**1) The study does not include a formal statistical power calculation since a feasibility study - and this is acceptable. However, it means the study is not powered to draw definitive conclusions about effectiveness and any effectiveness statistics need to be clearly presented as exploratory.**

**Response:**

We thank the reviewer for this important observation. We agree that, as a feasibility study, the primary objectives were to evaluate the feasibility, acceptability, usability, and preliminary outcomes of the AdaptRehab VR intervention rather than to establish definitive intervention effectiveness. Accordingly, this study was not designed or powered to detect statistically significant treatment effects but to estimate feasibility parameters and generate preliminary effect size estimates to inform the design of a future randomized controlled trial. We added a paragraph in the discussion to further highlight this important point (Discussion, p.35, lines 633-646).

**2) The manuscript does not clearly describe how missing data were handled. It's important to state whether analyses were based on complete cases only or if multiple imputation methods were considered if relevant.**

**Response:**
Missing data were manually reviewed prior to analysis, and only participants with available baseline and post-intervention assessments were included in the outcome analyses. Therefore, the outcome analyses were conducted using complete-case data. No post-intervention outcome data were missing among participants who completed the study; thus, multiple imputation was not required.

**3) The manuscript reports p-values clearly for the main outcomes and includes confidence intervals for some estimates. However, confidence intervals are not consistently presented across all results, and there is some reliance on p-values despite the small sample. More consistent reporting of effect sizes with confidence intervals would strengthen interpretation and better inform a future trial.**

**Response:**

You are right, thank you for noticing this. Although the preliminary results about efficacy are not the most important part of this study, given it’s a feasibility study, presenting the 95%CI consistently is important. In the revised manuscript, we have strengthened the reporting of quantitative outcomes by consistently presenting effect size estimates and 95% confidence intervals alongside p-values where applicable.

**We revised the methods section of the manuscript as follows:**

In addition, given the feasibility design and small sample size, the analyses were considered exploratory. Effect sizes and 95% confidence intervals were reported alongside p-values to facilitate interpretation of preliminary intervention effects.

**We also revised the results section of the manuscript as follows:**

The mean SUS total score was 76.2 ± 12.1 (range: 52.5–92.5), indicating good usability above the commonly accepted threshold of 68. The 95% confidence interval (CI) for the mean SUS score was 69.8–83.0, while the 95% bootstrap CI was 69.8–81.7, demonstrating the precision and robustness of the usability estimate [61]

The attractiveness score was 1.89 ± 0.67, indicating a positive user experience. The 95% CI for the mean score was 1.56–2.22, while the 95% bootstrap CI was 1.59–2.18. The Pragmatic Quality score (Perspicuity, Efficiency, and Dependability) was 1.69 ± 0.71, with a 95% CI of 1.34–2.04 and a 95% bootstrap CI of 1.43–1.96. The Hedonic Quality score (Stimulation and Novelty) was 1.74 ± 0.82, with a 95% CI of 1.33–2.15. and a 95% bootstrap CI of 1.39–2.09.

**4) A study protocol is provided, and the manuscript appears broadly consistent with in terms of key aspects. But the paper does not explicitly describe adherence to the protocol or report any deviations, where one would expect in the methods section of the paper.**

**Response:** We agree that adherence to the predefined protocol and reporting of any deviations should be clearly described in the manuscript. In the revised manuscript, we have added a subsection in the methods section to explicitly report protocol adherence and deviations.

**We added a new subsection entitled "Protocol Adherence and Deviations" to the Methods section, as follows:**

**Protocol Adherence and Deviations**

The study was conducted in accordance with the approved study protocol reviewed and approved by the Institutional Review Board (IRB) of Jimma University (JUIH/IRB/0479/25; approval date: 12 April 2025) and registered as a clinical trial (NCT07412899). The study procedures, including the study design, participant eligibility criteria, AdaptRehab VR intervention, and outcome assessments, were implemented as described in the predefined protocol.

**5) Analyses are based primarily on paired t‑tests and p-values, with no use of regression modelling. While this may be acceptable for a small feasibility study, it limits the ability to account for confounding or explore variability in outcomes. Consider using regression-based approaches (even simple models) or, at minimum, emphasising estimation (effect sizes and confidence intervals) rather than relying on p-values alone.**

**Response:**
We agree that regression-based approaches may provide additional insight into factors associated with outcome variability. However, given the feasibility nature of this study and the small sample size (n = 18), we considered multivariable regression modelling inappropriate because of the risk of unstable estimates and overfitting. In the revised manuscript, we have strengthened the statistical reporting by emphasizing estimation rather than statistical significance alone. Effect sizes and 95% confidence intervals have been added where applicable, and the interpretation of outcome analyses has been revised to clearly indicate that these findings are exploratory and intended to inform the design of a future adequately powered randomized controlled trial. Thus, regression modelling was considered inappropriate because the available sample size would not provide stable parameter estimates and would substantially increase the risk of model overfitting.

**Revision in methods section:**

In addition, in light of the feasibility design and small sample size, all analyses were considered exploratory. Effect sizes and 95% confidence intervals were reported alongside p-values to facilitate the interpretation of preliminary intervention effects.

**6) when reporting feasibility outcomes, it would make sense to report uncertainty around them, for example using bootstrap.**

**Response:**
We agree that reporting uncertainty estimates around feasibility outcomes provides a more informative interpretation, particularly in a small feasibility study.

**We are incorporated the comments in methods section:**

Bootstrap resampling procedures were applied to key feasibility outcomes to quantify uncertainty around feasibility estimates. Bootstrap confidence intervals (95%) were generated by repeatedly resampling participant-level data with replacement and calculating the distribution of the parameter estimates. This approach was selected because of the small sample size and its ability to provide robust estimates of uncertainty without relying on strict distributional assumptions.

**Revision in results section:**

The mean SUS total score was 76.2 ± 12.1 (range: 52.5–92.5), indicating good usability above the commonly accepted threshold of 68. The 95% confidence interval (CI) for the mean SUS score was 69.8–83.0, while the 95% bootstrap CI was 69.8–81.7, demonstrating the precision and robustness of the usability estimate [61]

The attractiveness score was 1.89 ± 0.67, indicating a positive user experience. The 95% CI for the mean score was 1.56–2.22, while the 95% bootstrap CI was 1.59–2.18. The Pragmatic Quality score (Perspicuity, Efficiency, and Dependability) was 1.69 ± 0.71, with a 95% CI of 1.34–2.04 and a 95% bootstrap CI of 1.43–1.96. The Hedonic Quality score (Stimulation and Novelty) was 1.74 ± 0.82, with a 95% CI of 1.33–2.15 and a 95% bootstrap CI of 1.39–2.09.

**7) the design limitations (no controls) need to make the authors more careful in their analyses in terms of effectiveness. I am also concerned about the lack of a control group, if the aim is to gauge effectiveness towards powering a larger study.**

**Response:**
We revised the discussion section of the paper incorporating the following points.

**We corrected as follows, in discussion:**

The absence of a control group is a key limitation of this feasibility study. Although participants demonstrated improvements following the AdaptRehab VR intervention, the single-arm pre–post design precludes causal inferences regarding intervention effectiveness. The observed changes may have been influenced by natural recovery, repeated testing, participant adaptation, or other uncontrolled factors. Consequently, these findings should be interpreted as preliminary evidence of feasibility and potential clinical benefit rather than definitive evidence of efficacy. A fully powered randomized controlled trial with an appropriate comparison group is warranted to establish the efficacy of AdaptRehab VR for upper limb stroke rehabilitation.

**Q#8: *Clarify if analysis is ITT or per protocol.***

**Response:**
Analyses were conducted using a per-protocol approach because the primary objective was to evaluate intervention feasibility among participants who completed the intervention. Recruitment, attrition and retention outcomes were reported for all enrolled participants.

**Q#9: analyses do not account for baseline values, which is expected for samples of this size. nevertheless perhaps this should be explored under a regression framework in terms of feasibility - or needs to be discussed as a potential limitation.**

**Response:**
Thank you for this suggestion. Given the small sample size and feasibility design, we used paired analyses rather than regression models.

## **Reviewer #2:**

**This study evaluates the feasibility, usability, acceptability, safety, and preliminary efficacy of the AdaptRehab VR system for upper-limb stroke rehabilitation in Ethiopia using a mixed-methods pre-post feasibility design. The topic is relevant for LMIC rehabilitation; however, several methodological and reporting weaknesses limit the strength of the conclusions.**

**Q#1: Provide a complete description of concurrent therapy.**

**Response:**
We thank the reviewer for raising this important issue. The concurrent therapy description is essential for improving transparency and supporting the interpretation of the study findings. We have revised the manuscript as follows in methods and discussion sections.

**Revision in methods**

As this was a feasibility study, participants were not restricted from receiving concurrent therapies during the study period, including conventional rehabilitation and routine stroke-related medical care. However, the specific types, frequencies, and durations of these additional interventions were not prospectively recorded.

**Revision in discussion section**

Therefore, concurrent therapies may have influenced participants' outcomes. Consequently, the observed improvements should be interpreted carefully, as the specific contribution of AdaptRehab VR cannot be fully distinguished from the effects of other ongoing rehabilitation interventions.

**Q#2: Add cognitive screening procedures and results.**

**Response:**
We acknowledge that pre-intervention cognitive screening was not conducted, which may have limited our ability to identify participants with cognitive difficulties affecting engagement with the VR intervention. We will add cognitive evaluation and screening for the RCT. One participant (P20) discontinued participation due to cognitive impairments that substantially limited their ability to understand instructions and engage with the intervention. We have added this limitation to the revised manuscript in discussion section and highlighted the importance of including standardized cognitive screening in future studies.

**Revision in discussion:**

Pre-intervention cognitive screening was not performed, which may have limited the identification of participants with cognitive difficulties affecting engagement. One participant (P20) discontinued due to cognitive impairments that limited their ability to understand instructions and interact with the AdaptRehab VR intervention. Future studies will incorporate standardized cognitive screening (Montreal Cognitive Assessment (MoCA) or the Mini-Mental State Examination (MMSE)) before enrolment.

**Q#3: Include feasibility progression criteria.**

**Response:**
We thank the reviewer for this important suggestion. We agree that predefined feasibility progression criteria are essential for interpreting the outcomes of a feasibility study. In the revised manuscript, we have added a feasibility progression criteria subsection in the methods section and discussed the implications of these findings in the discussion section.

**Revision in methods**

Feasibility progression criteria were defined a priori based on recommendations for pilot and feasibility studies [48], [59]. Criteria included recruitment, retention, adherence, usability, and safety outcomes. Progression to a larger trial was considered feasible if the intervention demonstrated acceptable retention (≥80%), adherence (≥80% of sessions), usability (SUS ≥70), and no serious intervention-related adverse events [60].

**Revision in discussion section**

The predefined feasibility criteria were achieved, with a retention rate of 90%, adherence of 93%, acceptable usability (SUS: 76.2 ± 12.1), and no serious intervention-related adverse events. These findings support the feasibility of progressing to a larger controlled trial, although further evaluation using a randomized controlled design is required to establish the intervention's effectiveness [59,66]. The high adherence rate further demonstrates the feasibility and acceptability of delivering imVR-based rehabilitation in this context.

**Q#4: Report confidence intervals consistently for all primary outcomes.**

**Response:**

In the revised manuscript, we have strengthened the reporting of quantitative outcomes by consistently presenting effect size estimates and 95% confidence intervals alongside p-values where applicable. To account for uncertainty associated with small sample size we reported uncertainty estimates using Bootstrap resampling procedures.

**Revision in result sections:**

The mean SUS total score was 76.2 ± 12.1 (range: 52.5–92.5), indicating good usability above the commonly accepted threshold of 68. The 95% confidence interval (CI) for the mean SUS score was 69.8–83.0, while the 95% bootstrap CI was 69.8–81.7, demonstrating the precision and robustness of the usability estimate [61], Fig 2A.

The attractiveness score was 1.89 ± 0.67, indicating a positive user experience. The 95% CI for the mean score was 1.56–2.22, while the 95% bootstrap CI was 1.59–2.18. The Pragmatic Quality score (Perspicuity, Efficiency, and Dependability) was 1.69 ± 0.71, with a 95% CI of 1.34–2.04 and a 95% bootstrap CI of 1.43–1.96. The Hedonic Quality score (Stimulation and Novelty) was 1.74 ± 0.82, with a 95% CI of 1.33–2.15 and a 95% bootstrap CI of 1.39–2.09 were both rated positively, indicating that participants found the system both usable and engaging. Complete results are presented in Fig 3.

**Q#5: How much conventional physiotherapy did each participant receive during the study period?**

**Response**: We have incorporated this comment into the revised manuscript as follows:

The participants received concurrent therapy during the study period, including conventional rehabilitation and routine stroke-related medical care. However, information regarding the specific types, frequencies, and durations of additional therapies was unfortunately not collected prospectively.

**Q#6: Was intervention intensity identical across participants?**

**Response**: This is indeed a very important point, while the duration was matched between patients we did not measure or evaluate the intensity. We will try to measure the intensity for the following RCT.

***Revision in methods section:***

The AdaptRehab VR intervention intensity was standardized across participants (9 sessions over 3 weeks, approximately 30 minutes per session). But variations in access to concurrent rehabilitation services may have influenced overall rehabilitation exposure and outcomes.

**Q#7: Were ARAT and BBT assessors independent from intervention delivery?**

**Response:**
We thank the reviewer for this important question. The ARAT and BBT assessments were conducted by trained assessors who were involved in the study assessments. However, because this was a feasibility pilot study with limited personnel resources, full assessor blinding was not feasible.

**Revision in methods section:**

The ARAT and BBT assessments were conducted by assessors who were independent of the intervention delivery. However, due to staffing limitations, the assessors were not blinded to participants' study participation.

**Q#8: Were minimal clinically important differences predefined?**

**Response:**
We thank the reviewer for this important suggestion. MCIDs were not predefined as progression criteria in this feasibility study. However, we have added a post hoc comparison of the observed changes with previously reported MCID values to provide clinical context for the findings.

**Revision in discussion section:**

Although Minimal clinically important differences (MCIDs) were not predefined in this feasibility study, the observed improvements provide preliminary evidence of potential clinical relevance. The mean ARAT improvement (15.6 points) exceeded the reported MCID of 5–6 points in chronic stroke survivors [69]. Similarly, the mean BBT improvement (8.7 blocks) exceeded the reported MCID of 5–6 blocks [70].

**Q#9: How were missing data handled for dropouts?**

**Response:**
**We have revised the methods section to clearly describe our approach to missing data.**

Missing data were manually reviewed prior to analysis, and only participants with available baseline and post-intervention assessments were included in the outcome analyses. Therefore, the outcome analyses were conducted using complete-case data. No post-intervention outcome data were missing among participants who completed the study; thus, multiple imputation was not required.

**Q#10: Why was a paired t-test selected without reporting normality testing?**

**Response:**
We thank the reviewer for this important comment. Normality was checked before applying the t-test using graphical methods (histogram, boxplot and q-q plot).

**Q#11: Was trial registration completed prospectively before recruitment?**

**Response:**
The study protocol was approved by the Institutional Review Board of Jimma University (JUIH/IRB/0479/25; approval date: 12 April 2025) before participant recruitment commenced. The study was prospectively registered as a clinical trial (NCT07412899). Participant recruitment started on 1 September 2025 and was completed on 10 October 2025.

**Q#12: What motor-learning principles were explicitly embedded in AdaptRehab VR (task specificity, variability, knowledge of results, knowledge of performance)?**

**Response:**We agree that describing the motor-learning principles embedded in the AdaptRehab VR intervention improves the understanding of its rehabilitation rationale.

Task specificity was incorporated through goal-directed upper limb activities that simulated functional reaching, grasping, and object manipulation tasks. Practice variability was included by providing different game scenarios and task challenges to promote adaptation and motor skill acquisition. Knowledge of results was provided through immediate feedback on task performance, scores, and completion status, while knowledge of performance was supported through visual guidance and interaction feedback during task execution. These principles were incorporated to promote active engagement, repetitive practice, and motor learning during rehabilitation.

**Q#13: How was movement quality quantified beyond ARAT/BBT outcomes?**

**Response:**
Movement quality was not quantified using additional kinematic or sensor-based measures beyond the ARAT and BBT in this feasibility study. We believe that these clinical outcome measures were sufficient for assessing preliminary feasibility.

**Q#14: Does the system adapt difficulty automatically or manually? Provide the adaptation logic.**

**Response:**
The system did not include an automatic difficulty adaptation algorithm. The difficulty level of VR tasks was adjusted manually by rehabilitation professionals based on each participant’s functional ability, performance, and clinical status. Adaptation decisions were guided by therapists’ recommendations rather than an automated algorithm.

**Q#15: Were ceiling/floor effects assessed for ARAT and BBT?**

**Response:**
We have acknowledged this points but ceiling and floor effects were not formally assessed due to the feasibility nature and small sample size of this pilot study.

**Q#16: What proportion of improvement exceeded measurement error and MDC values?**

**Response:**
As MDC values require established reliability estimates or validated thresholds from the literature ( see discussion section), this analysis was beyond the scope of the current pilot study. **Revision in discussion section:**

Although minimal clinically important differences (MCIDs) were not predefined in this feasibility study, the observed improvements provide preliminary evidence of potential clinical relevance. The ARAT improvements (15.6 points) exceeded the MCID of 5-6 points in chronic stroke survivors [72], and BBT improvements (8.7 blocks) also exceeded the MCID of 5-6 blocks [73].

## **Reviewer #3:**

**1- The research contributions are explained in concise bullet points, with numerical values added for the adopted metrics in a paragraph within the introduction. Additionally, another paragraph at the end of the introduction outlines the structure of the manuscript in its entirety.**

**Response:**
Thank you for taking the time to read this manuscript and for your valuable suggestions. The most important numbers (i.e., incidence, impact for the patients) are presented in the introduction to highlight the challenge and severity of stroke management, particularly in LMICs.

**2- Figure 6 separates branches A and B into two figures to make them clearer.**

We created two separate figures to ease the readability and interpretation.

**3- The conclusions are written briefly and are merely a narrative without any numerical values or reference to any figures or tables used in the results. Furthermore, the proposed future work is unclear.**

Regarding the request to include numerical values and figure/table references in the Conclusions, we would respectfully offer the following clarification. In accordance with standard scientific reporting conventions the conclusions section is intended to synthesize the overarching implications and take-home messages of the study, rather than to reiterate specific quantitative results. Detailed numerical data, statistical outputs, and references to specific figures and tables are appropriately placed in the Results and Discussion sections, where they are contextualized and interpreted. We have therefore retained the descriptive synthesis in the Conclusions while ensuring that all numerical evidence is fully presented and discussed in the preceding sections.

We do, however, fully agree with your second point: the proposed future work was indeed vague and lacked clear direction. To address this, we have substantially revised the Conclusions to provide a structured and actionable research agenda. Specifically, we now clearly outline the following priorities: (1) a definitive randomized controlled trial with long-term follow-up; (2) implementation science research to address practical barriers; (3) cost-effectiveness analyses; (4) exploration of home-based delivery models; and (5) ongoing cultural adaptation. We have also explicitly linked these future directions to challenges identified during the study (e.g., transportation barriers, cognitive screening needs, space constraints), ensuring that the roadmap is grounded in our actual findings.
