# Supplementary material for: Feasibility, acceptability, and preliminary efficacy of AdaptRehab VR, an immersive virtual reality system for upper limb stroke rehabilitation: A mixed-methods study in Ethiopia
Source: PLoS One. 2026 Oct 9;21(10):e0360216. doi: 10.1371/journal.pone.0360216 (PMC13652278; doi:10.1371/journal.pone.0360216)
Supplement: Peer review history

# Author comment: Author response to Decision Letter 0

**DOI:** 10.1371/journal.pone.0360216.r001

**Transfer Alert**

This paper was transferred from another journal. As a result, its full editorial history (including decision letters, peer reviews and author responses) may not be present.

27 May 2026

# Peer review: Decision Letter 0

**DOI:** 10.1371/journal.pone.0360216.r002

**Authors:** Yuk Ming Tang

2 Jul 2026

Dear Dr. Kenea,

Thank you for submitting your manuscript to PLOS ONE. After careful consideration, we feel that it has merit but does not fully meet PLOS ONE’s publication criteria as it currently stands. Therefore, we invite you to submit a revised version of the manuscript that addresses the points raised during the review process.

Please submit your revised manuscript by Aug 31 2026 11:59PM. If you will need more time than this to complete your revisions, please reply to this message or contact the journal office at plosone@plos.org. When you're ready to submit your revision, log on to https://www.editorialmanager.com/pone/ and select the 'Submissions Needing Revision' folder to locate your manuscript file.

- A letter that responds to each point raised by the academic editor and reviewer(s). You should upload this letter as a separate file labeled 'Response to Reviewers'.
- A marked-up copy of your manuscript that highlights changes made to the original version. You should upload this as a separate file labeled 'Revised Manuscript with Track Changes'.
- An unmarked version of your revised paper without tracked changes. You should upload this as a separate file labeled 'Manuscript'.

If you would like to make changes to your financial disclosure, please include your updated statement in your cover letter. Guidelines for resubmitting your figure files are available below the reviewer comments at the end of this letter.

If applicable, we recommend that you deposit your laboratory protocols in protocols.io to enhance the reproducibility of your results. Protocols.io assigns your protocol its own identifier (DOI) so that it can be cited independently in the future. For instructions see: https://journals.plos.org/plosone/s/submission-guidelines#loc-laboratory-protocols. Additionally, PLOS ONE offers an option for publishing peer-reviewed Lab Protocol articles, which describe protocols hosted on protocols.io. Read more information on sharing protocols at https://plos.org/protocols?utm_medium=editorial-email&utm_source=authorletters&utm_campaign=protocols.

As the corresponding author, your ORCID iD is verified in the submission system and will appear in the published article. PLOS supports the use of ORCID, and we encourage all coauthors to register for an ORCID iD and use it as well. Please encourage your coauthors to verify their ORCID iD within the submission system before final acceptance, as unverified ORCID iDs will not appear in the published article. *Only*  the individual author can complete the verification step; PLOS staff *cannot*  verify ORCID iDs on behalf of authors.

We look forward to receiving your revised manuscript.

Kind regards,

Yuk Ming Tang

Academic Editor

PLOS One

Journal Requirements:

When submitting your revision, we need you to address these additional requirements.

1. Please ensure that your manuscript meets PLOS ONE's style requirements, including those for file naming. The PLOS ONE style templates can be found at

https://journals.plos.org/plosone/s/file?id=wjVg/PLOSOne_formatting_sample_main_body.pdf and

https://journals.plos.org/plosone/s/file?id=ba62/PLOSOne_formatting_sample_title_authors_affiliations.pdf

2. Please include a complete copy of PLOS’ questionnaire on inclusivity in global research in your revised manuscript. Our policy for research in this area aims to improve transparency in the reporting of research performed outside of researchers’ own country or community. The policy applies to researchers who have travelled to a different country to conduct research, research with Indigenous populations or their lands, and research on cultural artefacts. The questionnaire can also be requested at the journal’s discretion for any other submissions, even if these conditions are not met.  Please find more information on the policy and a link to download a blank copy of the questionnaire here: https://journals.plos.org/plosone/s/best-practices-in-research-reporting. Please upload a completed version of your questionnaire as Supporting Information when you resubmit your manuscript.

3. Please note that PLOS One has specific guidelines on code sharing for submissions in which author-generated code underpins the findings in the manuscript. In these cases, we expect all author-generated code to be made available without restrictions upon publication of the work. Please review our guidelines at https://journals.plos.org/plosone/s/materials-and-software-sharing#loc-sharing-code and ensure that your code is shared in a way that follows best practice and facilitates reproducibility and reuse.

4. In the online submission form, you indicated that [The datasets used and/or analysed during the current study are available from the corresponding author on reasonable request].

All PLOS journals now require all data underlying the findings described in their manuscript to be freely available to other researchers, either 1. In a public repository, 2. Within the manuscript itself, or 3. Uploaded as supplementary information.

This policy applies to all data except where public deposition would breach compliance with the protocol approved by your research ethics board. If your data cannot be made publicly available for ethical or legal reasons (e.g., public availability would compromise patient privacy), please explain your reasons on resubmission and your exemption request will be escalated for approval.

5. Your ethics statement should only appear in the Methods section of your manuscript. If your ethics statement is written in any section besides the Methods, please delete it from any other section

6. If the reviewer comments include a recommendation to cite specific previously published works, please review and evaluate these publications to determine whether they are relevant and should be cited. There is no requirement to cite these works unless the editor has indicated otherwise.

[Note: HTML markup is below. Please do not edit.]

Reviewers' comments:

Reviewer's Responses to Questions

**Comments to the Author**

1. Is the manuscript technically sound, and do the data support the conclusions?

Reviewer #1: Yes

Reviewer #2: No

Reviewer #3: Yes

**********

2. Has the statistical analysis been performed appropriately and rigorously?

Reviewer #1: Yes

Reviewer #2: No

Reviewer #3: Yes

**********

3. Have the authors made all data underlying the findings in their manuscript fully available?

The PLOS Data policy

Reviewer #1: Yes

Reviewer #2: Yes

Reviewer #3: Yes

**********

4. Is the manuscript presented in an intelligible fashion and written in standard English?

Reviewer #1: Yes

Reviewer #2: No

Reviewer #3: Yes

**********

Reviewer #1: As the statistical reviewer I will focus on methods and reporting.

Major

1) The study does not include a formal statistical power calculation since a feasibility study - and this is acceptable. However, it means the study is not powered to draw definitive conclusions about effectiveness and any effectiveness statistics need to be clearly presented as exploratory.

2) The manuscript does not clearly describe how missing data were handled. It's important to state whether analyses were based on complete cases only or if multiple imputation methods were considered if relevant.

3) The manuscript reports p-values clearly for the main outcomes and includes confidence intervals for some estimates. However, confidence intervals are not consistently presented across all results, and there is some reliance on p-values despite the small sample. More consistent reporting of effect sizes with confidence intervals would strengthen interpretation and better inform a future trial.

4) A study protocol is provided, and the manuscript appears broadly consistent with in terms of key aspects. But the paper does not explicitly describe adherence to the protocol or report any deviations, where one would expect in the methods section of the paper.

5) Analyses are based primarily on paired t‑tests and p-values, with no use of regression modelling. While this may be acceptable for a small feasibility study, it limits the ability to account for confounding or explore variability in outcomes. Consider using regression-based approaches (even simple models) or, at minimum, emphasising estimation (effect sizes and confidence intervals) rather than relying on p-values alone.

6) when reporting feasibility outcomes, it would make sense to report uncertainty around them, for example using bootstrap.

7) the design limitations (no controls) need to make the authors more careful in their analyses in terms of effectiveness. I am also concerned about the lack of a control group, if the aim is to gauge effectiveness towards powering a larger study.

Minor

1) clarify if analysis is ITT or per protocol

2) analyses do not account for baseline values, which is expected for samples of this size. nevertheless perhaps this should be explored under a regression framework in terms of feasibility - or needs to be discussed as a potential limitation.

Reviewer #2: This study evaluates the feasibility, usability, acceptability, safety, and preliminary efficacy of the AdaptRehab VR system for upper-limb stroke rehabilitation in Ethiopia using a mixed-methods pre-post feasibility design. The topic is relevant for LMIC rehabilitation; however, several methodological and reporting weaknesses limit the strength of the conclusions.

Provide a complete description of concurrent therapy.

Add cognitive screening procedures and results.

Include feasibility progression criteria.

Report confidence intervals consistently for all primary outcomes.

Expand limitations and discuss sources of bias more critically.

Provide a stronger justification for generalizability to LMIC settings.

Include a detailed technical description of AdaptRehab VR and its adaptive mechanisms.

How much conventional physiotherapy did each participant receive during the study period?

Was intervention intensity identical across participants?

Were ARAT and BBT assessors independent from intervention delivery?

Were minimal clinically important differences predefined?

How were missing data handled for dropouts?

Why was a paired t-test selected without reporting normality testing?

Was trial registration completed prospectively before recruitment?

What motor-learning principles were explicitly embedded in AdaptRehab VR (task specificity, variability, knowledge of results, knowledge of performance)?

How was movement quality quantified beyond ARAT/BBT outcomes?

Does the system adapt difficulty automatically or manually? Provide the adaptation logic.

Were ceiling/floor effects assessed for ARAT and BBT?

What proportion of improvement exceeded measurement error and MDC values?

Reviewer #3: 1- The research contributions are explained in concise bullet points, with numerical values added for the adopted metrics in a paragraph within the introduction. Additionally, another paragraph at the end of the introduction outlines the structure of the manuscript in its entirety.

2- Figure 6 separates branches A and B into two figures to make them clearer.

3- The conclusions are written briefly and are merely a narrative without any numerical values or reference to any figures or tables used in the results. Furthermore, the proposed future work is unclear.

**********

what does this mean?). If published, this will include your full peer review and any attached files.

If you choose “no”, your identity will remain anonymous but your review may still be made public.

**Do you want your identity to be public for this peer review?**  For information about this choice, including consent withdrawal, please see our Privacy Policy

Reviewer #1: No

Reviewer #2: No

Reviewer #3: No

**********

[NOTE: If reviewer comments were submitted as an attachment file, they will be attached to this email and accessible via the submission site. Please log into your account, locate the manuscript record, and check for the action link "View Attachments". If this link does not appear, there are no attachment files.]

To ensure your figures meet our technical requirements, please review our figure guidelines: https://journals.plos.org/plosone/s/figures

You may also use PLOS’s free figure tool, NAAS, to help you prepare publication quality figures: https://journals.plos.org/plosone/s/figures#loc-tools-for-figure-preparation.

NAAS will assess whether your figures meet our technical requirements by comparing each figure against our figure specifications.

# Author comment: Author response to Decision Letter 1

**DOI:** 10.1371/journal.pone.0360216.r003

21 Aug 2026

Thank you very much for your time and for providing encouraging and constructive comments. We have carefully considered each suggestion and revised the manuscript accordingly. We have addressed and incorporated all comments, questions, and suggestions from the reviewers and editors, and the manuscript has been revised accordingly. Detailed responses to each comment, along with the corresponding revisions made in the manuscript, are provided in the attached “Response to Reviewers” document.

Attachment Submitted filename: Response to Reviewers_Final_BB.docx

# Peer review: Decision Letter 1

**DOI:** 10.1371/journal.pone.0360216.r004

**Authors:** Yuk Ming Tang

14 Sep 2026

Feasibility, Acceptability, and Preliminary Efficacy of AdaptRehab VR, an Immersive Virtual Reality System for Upper Limb Stroke Rehabilitation: A Mixed-Methods Study in Ethiopia

PONE-D-26-23266R1

Dear Dr. Kenea,

We’re pleased to inform you that your manuscript has been judged scientifically suitable for publication and will be formally accepted for publication once it meets all outstanding technical requirements.

Within one week, you’ll receive an e-mail detailing the required amendments. When these have been addressed, you’ll receive a formal acceptance letter and your manuscript will be scheduled for publication.

An invoice will be generated when your article is formally accepted. Please note, if your institution has a publishing partnership with PLOS and your article meets the relevant criteria, all or part of your publication costs will be covered. Please make sure your user information is up-to-date by logging into Editorial Manager at Editorial Manager® and clicking the ‘Update My Information' link at the top of the page. For questions related to billing, please contact billing support.

If your institution or institutions have a press office, please notify them about your upcoming paper to help maximize its impact. If they’ll be preparing press materials, please inform our press team as soon as possible -- no later than 48 hours after receiving the formal acceptance. Your manuscript will remain under strict press embargo until 2 pm Eastern Time on the date of publication. For more information, please contact onepress@plos.org.

Kind regards,

Yuk Ming Tang

Academic Editor

PLOS One

Additional Editor Comments (optional):

Reviewers' comments:

Reviewer's Responses to Questions

**Comments to the Author**

Reviewer #1: All comments have been addressed

Reviewer #2: (No Response)

**********

2. Is the manuscript technically sound, and do the data support the conclusions?

Reviewer #1: Yes

Reviewer #2: (No Response)

**********

3. Has the statistical analysis been performed appropriately and rigorously?

Reviewer #1: Yes

Reviewer #2: (No Response)

**********

4. Have the authors made all data underlying the findings in their manuscript fully available?

The PLOS Data policy

Reviewer #1: Yes

Reviewer #2: (No Response)

**********

5. Is the manuscript presented in an intelligible fashion and written in standard English?

Reviewer #1: Yes

Reviewer #2: (No Response)

**********

Reviewer #1: I am satisfied with the authors' responses and the resulting changes to the paper. I have nothing else to add.

Reviewer #2: (No Response)

**********

what does this mean?). If published, this will include your full peer review and any attached files.

If you choose “no”, your identity will remain anonymous but your review may still be made public.

**Do you want your identity to be public for this peer review?**  For information about this choice, including consent withdrawal, please see our Privacy Policy

Reviewer #1: No

Reviewer #2: No

**********

# Editor report: Acceptance letter

**DOI:** 10.1371/journal.pone.0360216.r005

**Authors:** Yuk Ming Tang

PONE-D-26-23266R1

PLOS One

Dear Dr. Kenea,

I'm pleased to inform you that your manuscript has been deemed suitable for publication in PLOS One. Congratulations! Your manuscript is now being handed over to our production team.

At this stage, our production department will prepare your paper for publication. This includes ensuring the following:

* All references, tables, and figures are properly cited

* All relevant supporting information is included in the manuscript submission,

* There are no issues that prevent the paper from being properly typeset

You will receive further instructions from the production team, including instructions on how to review your proof when it is ready. Please keep in mind that we are working through a large volume of accepted articles, so please give us a few days to review your paper and let you know the next and final steps.

Lastly, if your institution or institutions have a press office, please let them know about your upcoming paper now to help maximize its impact. If they'll be preparing press materials, please inform our press team within the next 48 hours. Your manuscript will remain under strict press embargo until 2 pm Eastern Time on the date of publication. For more information, please contact onepress@plos.org.

You will receive an invoice from PLOS for your publication fee after your manuscript has reached the completed accept phase. If you receive an email requesting payment before acceptance or for any other service, this may be a phishing scheme. Learn how to identify phishing emails and protect your accounts at https://explore.plos.org/phishing.

If we can help with anything else, please email us at customercare@plos.org.

Thank you for submitting your work to PLOS One and supporting open access.

Kind regards,

PLOS One Editorial Office Staff

on behalf of

Dr. Yuk Ming Tang

Academic Editor

PLOS One
